# From healing landscapes to biocultural conservation: the role of *Ficus septica* in karst ecosystems of South Sulawesi, Indonesia

**DOI:** 10.1186/s13002-026-00896-3

**Published:** 2026-04-04

**Authors:** Ahmad Ismail, Eymal B. Demmallino, Yohannes Purwanto, Indra A. S. L. P. Putri, Andi Batara Al Isra, Taqiyyah Al Ghumaidha Alta

**Affiliations:** 1 Department of Anthropology, Faculty of Social and Political Sciences, Hasanuddin University, Makassar, 90245 Indonesia; 2 Department of Agricultural Socioeconomics, Faculty of Agriculture, Hasanuddin University, Makassar, 90245 Indonesia; 3 Research Center for Ecology and Ethnobiology, National Research and Innovation Agency (BRIN), Jalan Raya Jakarta-Bogor Km 46, Cibinong, 16911 Indonesia; 4 Department of Biology, Faculty of Exact Sciences and Natural Sciences, Hasanuddin University, Makassar, 90245 Indonesia

**Keywords:** *Ficus septica*, Biocultural conservation, Ethnographic, Karst ecosystem restoration, Community-based conservation, Traditional ecological knowledge, Indonesia

## Abstract

**Background:**

Biocultural landscapes in Indonesia preserve extensive medicinal plant knowledge, yet many species remain insufficiently examined through integrative ethnographic, ecological, and phytochemical approaches. Among the Bugis–Makassar communities of South Sulawesi, *Ficus septica* (tobo-tobo/awar-awar) is regarded as a “cooling” plant used to treat heat-related disorders and restore *assitinajang* (cosmic balance). However, limited research has explored how its cultural classifications correspond to phytochemical properties and how its presence relates to ecological dynamics in karst environments.

**Methods:**

This study combined in-depth ethnographic interviews, participant observation, and ethnobotanical documentation with Gas Chromatography–Mass Spectrometry (GC–MS) profiling of methanolic leaf extracts of *F. septica* collected in Balleanging Village, Pangkep Regency. Five purposively selected knowledge holders, including traditional healers and elders, provided detailed narratives on plant classification, preparation techniques, and cosmological meanings. Ecological observations documented the species’ distribution patterns and associated plant–animal interactions within limestone habitats.

**Results:**

Participants consistently classified *F. septica* as a *penawar* (“cooling medicine”) that neutralizes *panasa’* (heat imbalance) and strengthens *sumange’* (vital force). GC–MS profiling identified fifty-four compounds, dominated by phytol, myristic acid vinyl ester, methyl palmitate, methyl stearate, and rotundifuran—molecules with documented anti-inflammatory, antioxidant, and antimicrobial activities. These pharmacological properties align with local thermal classifications, suggesting a meaningful correspondence between indigenous medical concepts and biochemical evidence. Ecological observations and supporting literature indicate that *F. septica* exhibits traits consistent with pioneer species in karst substrates and participates in frugivore-mediated seed dispersal networks.

**Conclusion:**

The convergence of cultural interpretation, phytochemical profiling, and ecological observation suggests that *F. septica* operates within a localized biocultural system in which healing practices, cosmological ethics, and ecological processes are interconnected. While based on a focused qualitative sample and site-specific ecological documentation, this study highlights the value of integrating traditional ecological knowledge with scientific inquiry to inform culturally grounded conservation strategies in karst landscapes.

**Supplementary Information:**

The online version contains supplementary material available at 10.1186/s13002-026-00896-3.

## Introduction

Traditional medicinal knowledge in Indonesia is embedded within one of the world’s most biologically and culturally diverse regions [1–8]. As a recognized megadiverse country, Indonesia hosts approximately 10% of the world’s flowering plant species and represents one of the highest levels of ethnolinguistic diversity globally, with more than 700 local languages and associated knowledge systems [4, 6, 9–11]. Despite this extraordinary biodiversity and cultural plurality, large portions of Indonesia’s ethnomedicinal knowledge remain insufficiently documented or examined through integrative scientific approaches that combine ethnography, ecology, and phytochemistry ([12, 13].

Ethnobotanical scholarship has repeatedly demonstrated that traditional medical systems often encode empirically grounded ecological observations, yet the systematic validation of such knowledge remains uneven across regions [14]. In Indonesia, where ecological complexity intersects with rapid socio-environmental change, the erosion of medicinal plant knowledge poses risks not only to cultural heritage but also to biodiversity conservation and community health resilience. Thus, advancing interdisciplinary research that bridges local epistemologies with biochemical and ecological analysis is essential for strengthening biocultural conservation frameworks.

Within this broader context of high biodiversity but uneven scientific documentation, Indonesia’s traditional medicinal systems can be understood not merely as local health practices but as extensive repositories of biocultural heritage. Across the archipelago’s diverse ethnic landscapes, local communities uphold intricate healing systems that integrate empirical experience with cosmological understanding. These systems embody centuries of accumulated ecological wisdom, wherein human health, spiritual balance, and environmental integrity are perceived as interdependent components of a singular living continuum. However, despite their persistence in daily practice, many indigenous pharmacopoeias remain under-documented and rarely validated through interdisciplinary research that bridges ethnography, chemistry, and ecology [15, 16]. This gap limits our understanding of how traditional healing frameworks correspond with biomedical and ecological evidence.

Traditional healing practices are not merely pragmatic responses to illness but also expressions of cosmological order—a worldview in which plants are imbued with vitality and agency. Within such systems, health is understood as the restoration of harmony between the human body, the natural environment, and the spiritual world. As argued by [17], indigenous ecological knowledge must be recognized as both cultural heritage and an environmental management practice, capable of informing sustainable conservation strategies. In Indonesia, where linguistic and ecological diversity intersect, the erosion of traditional knowledge due to modernization and habitat loss poses an immediate threat not only to cultural identity but also to biodiversity itself. Thus, the preservation of medicinal plant knowledge is inseparable from biocultural conservation efforts—protecting both living knowledge systems and the ecosystems that sustain them. Within this broader framework, the genus *Ficus* (Moraceae) plays a significant role across Southeast Asia [18–23]. Among its many species, *F. septica* Burm.f.—locally known as *awar-awar*,* tobo-tobo*, or *daussalo*—holds a distinctive position in the healing traditions and cosmological narratives of Indonesian, Specially in Bugis-Makassar communities. Beyond its practical use for treating fever, inflammation, and wounds [24, 25], *F. septica* embodies a moral concept of balance and vitality.

The Bugis–Makassar are two closely related Austronesian-speaking ethnic groups indigenous to South Sulawesi, Indonesia, historically known for maritime trade, complex social hierarchies, and rich cosmological traditions preserved in oral and written epics such as the *Sureq Galigo* [26, 27]. Within their moral–cosmological worldview, humans, plants, animals, and spiritual forces are understood as interconnected through shared vitality. Among the Bugis–Makassar people of South Sulawesi, *F. septica* is believed to possess *sumange’*, a vital force connecting human and non-human worlds through shared cosmic energy. This notion reflects a holistic philosophy in which illness arises from an imbalance of forces, and healing restores alignment between the moral, spiritual, and ecological realms. In Bugis–Makassar ethnomedicine, illness is commonly classified through a relational framework of “heat” (*panasa’*) and “coolness” (*penawar*), referring not merely to temperature but to embodied states of imbalance affecting physical, emotional, and moral well-being. Healing practices aim to restore equilibrium by counteracting excess “heat” and stabilizing the vital force (*sumange’*).

Ecologically, *F. septica* functions as a pioneer species in disturbed and limestone-based environments, stabilizing fragile karst soils and supporting frugivorous fauna [28]. Its ecological resilience mirrors its cultural role: a plant that both heals and regenerates. Yet, despite its prominence, few studies have integrated *F. septica*’s pharmacological, ecological, and cosmological dimensions within a single analytical framework.

Despite increasing pharmacological and ethnobotanical studies on *F. septica* across Southeast Asia, most previous research has examined the species through a single disciplinary lens—either focusing on isolated phytochemical compounds or documenting medicinal uses without ecological contextualization [29–32]. Few studies have integrated ethnographic interpretation, chemical profiling, and ecological observation within a unified analytical framework [33–35]. As a result, the relationship between local medical classifications, biochemical properties, and landscape-level ecological dynamics remains insufficiently explored.

This study addresses that gap by adopting an explicitly integrative biocultural approach. Rather than treating cultural belief, pharmacological evidence, and ecological presence as separate domains, we analyze *F. septica* as a species situated at the intersection of healing practice, cosmological meaning, and habitat resilience in a karst landscape. The novelty of this research lies not in introducing new pharmacological assays, but in synthesizing ethnographic depth, GC–MS phytochemical profiling, and ecological field observation into a single interpretive framework grounded in biocultural theory [13, 36].

In this study, key analytical terms are used in clearly defined ways. The designation of *F. septica* as a “cooling plant” refers to an emic thermal classification within Bugis–Makassar ethnomedicine, where conditions described as *panasa’* (heat imbalance) correspond to symptoms associated with inflammation, fever, and physiological agitation. The term “biocultural keystone species” is employed heuristically, following [36], to denote a species of simultaneous cultural salience and ecological relevance within a specific socio-ecological setting. This usage does not imply quantitative testing of keystone ecological metrics, but rather highlights the species’ combined symbolic prominence, medicinal centrality, and observed ecological functions in the Maros–Pangkep karst context. It seeks to answer the following questions:

1. How does *F. septica* embody the moral and cosmological principles of healing in Bugis–Makassar ethnomedical practice?
2. How do its phytochemical properties correspond to local classifications of “heat” and “coolness”?
3. How can this integration of cultural interpretation and ecological observation infrom participatory biocultural conservation strategies in karst landscapes?

## Materials and methods

### Study area

**Fig. 1:**
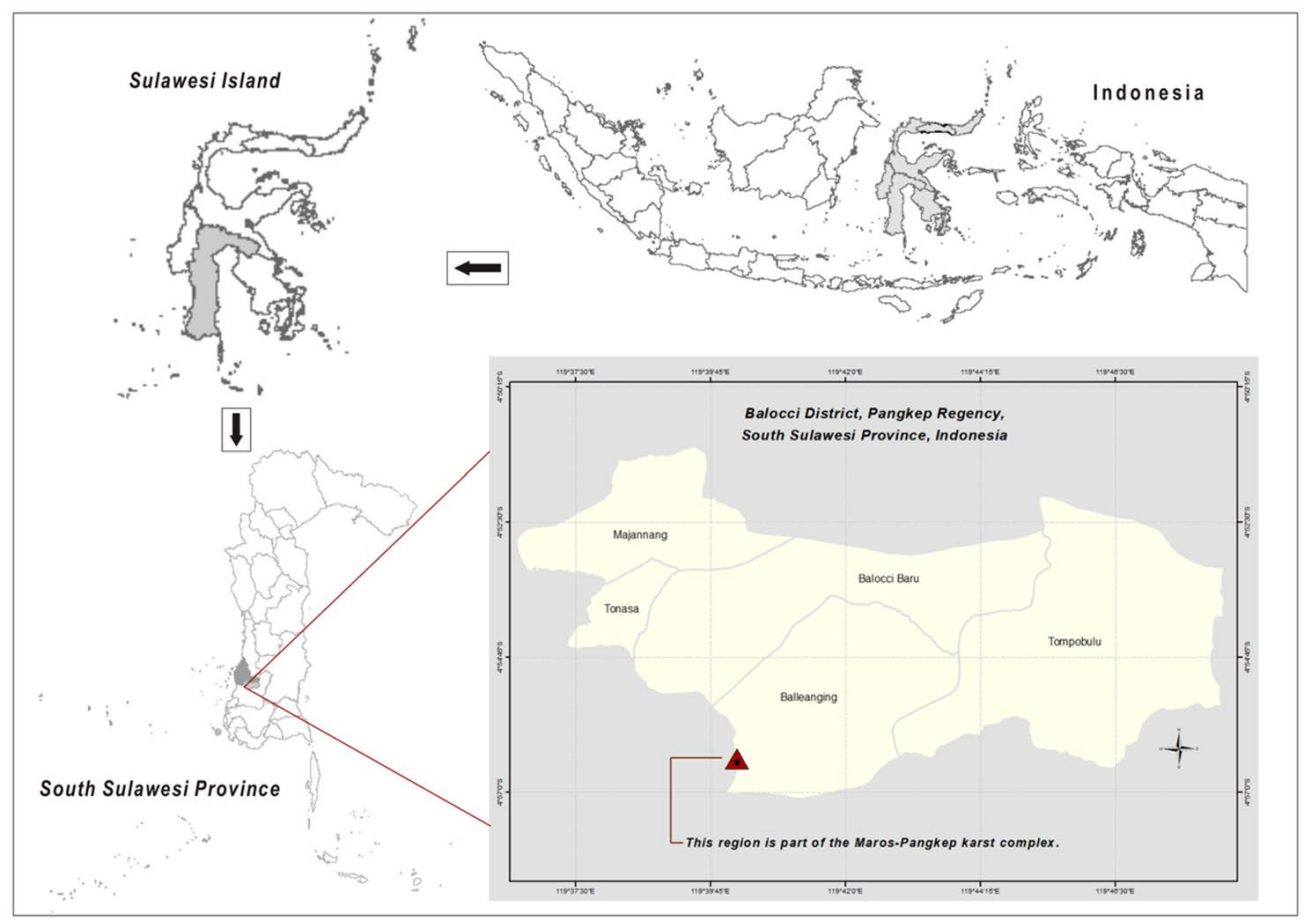
Location of the study area in Balleanging Village, Balocci District, Pangkep Regency, South Sulawesi Province, Indonesia. The inset maps show the geographical position of Sulawesi Island within the Indonesian archipelago and the provincial location of South Sulawesi. The main map illustrates Balleanging and its neighboring settlements—Tonasa, Majannang, Balocci Baru, and Tompobulu—situated within the Balocci District. The red triangle marks the field site where ethnobotanical and ecological data on *F. septica* were collected. This region forms part of the Maros–Pangkep karst complex, one of Indonesia’s largest limestone formations, characterized by rugged topography, thin soils, and unique ecological niches supporting high biocultural diversity

The research was conducted in Balleanging Village, located in Balocci District, Pangkep Regency, South Sulawesi Province, Indonesia (Fig. 1). Geographically, the site lies within the Maros–Pangkep karst complex, one of the largest continuous limestone landscapes in Southeast Asia, situated approximately 90 km north of Makassar. The karst terrain is characterized by steep limestone hills, sinkholes, subterranean rivers, and pockets of fertile alluvial soil that support limited but diverse vegetation. Elevations in the study area range from 70 m to 140 m above sea level, with an average annual temperature of 27–30 °C and rainfall exceeding 2,500 mm per year, typical of humid tropical conditions.

The site was purposively selected for three primary reasons. First, Balleanging lies within the Maros–Pangkep karst complex, allowing examination of plant–landscape relationships in a limestone ecosystem context. Second, *F. septica* is locally abundant and readily accessible across settlement margins and rocky slopes, making it suitable for combined ethnobotanical and ecological observation. Third, traditional healing practices involving this species remain actively transmitted among community members, providing a relevant socio-cultural setting for biocultural analysis.

Ecologically, the Maros–Pangkep karst functions as an important biodiversity refuge and hydrological buffer for surrounding lowland areas. Despite its fragility, the ecosystem sustains a mosaic of vegetation types, including secondary forests, home gardens, bamboo groves, and medicinal plant patches. Ficus species, particularly *F. septica*, thrive along limestone slopes and drainage channels where moisture and light conditions favor rapid regeneration. The plant’s adaptability to thin calcareous soils and its ecological role as a pioneer species contribute to local ecosystem stability, while also ensuring its constant availability for traditional healing purposes.

## Data collection

### Population and sample

This study combined ethnobotanical and ethnographic approaches with laboratory-based phytochemical analysis to explore the cultural, ecological, and biochemical significance of *F. septica* in the karst landscapes of South Sulawesi. Data were collected from June to September 2025 through semi-structured interviews, participant observation, and free-listing techniques.

Free-listing was used descriptively to document the range of medicinal plants recognized by informants and to identify the relative prominence of *F. septica* within local pharmacopoeia. No formal cultural salience index or quantitative ranking analysis was applied; instead, recurrent mentions across interviews were interpreted qualitatively to indicate relative importance within the interviewed group.

A total of five key informants were purposively selected from approximately 12 households in Balleanging Village and its surrounding hamlets. The selection included two traditional healers (*sanro*), two elderly knowledge holders, and one community member recognized for expertise in medicinal plants (Table 1). The inclusion criteria were based on intergenerational reputation, practical experience with herbal preparation, and participation in community healing practices.

Interviews were conducted in Bugis and Makassar and subsequently translated into Indonesian and English to ensure analytical clarity. Initial translations into Indonesian were carried out by bilingual researchers familiar with local terminology and healing practices, and were later cross-checked within the research team prior to English rendering to maintain semantic consistency. Particular attention was given to culturally specific concepts such as *panasa’* (heat imbalance), *sumange’* (vital force), and *assitinajang* (cosmic balance), whose meanings are embedded in local cosmology and cannot be reduced to direct biomedical equivalents. Key interview questions addressed: [1] local terminology and identification of *F. septica*; [2] traditional modes of preparation and application; [3] perceived therapeutic effects and classifications of illness, especially distinctions between “heat” and “cooling” conditions; and [4] symbolic meanings and cosmological interpretations associated with vitality, balance, and moral–ecological order.

Participant observation accompanied informants during plant collection, ritual preparation, and healing sessions, allowing detailed documentation of embodied practices and ecological context. All interactions followed ethical research protocols established by the Human Research Ethics Committee, Faculty of Social and Political Sciences, Universitas Hasanuddin, in accordance with the International Society of Ethnobiology [37]. Informed consent was obtained prior to each engagement, and community acknowledgment was ensured by written.

**Table 1:** Demographic profile of key informants involved in the ethnobotanical study of *F. septica* in Balleanging Village, South Sulawesi

Informant code	Gender	Age (years)	Social role	Primary expertise	Language used	Notes on participation
INF-01	Female	63	Traditional healer (*sanro*)	Preparation of herbal remedies for fever and inflammation	Bugis	Performed healing demonstration and ritual use of *F. septica*
INF-02	Male	71	Elder (*to matoa*)	Local taxonomy and symbolic plant classification	Bugis	Provided cosmological interpretation (*sumange’*)
INF-03	Female	58	Community herbalist	Plant identification and dosage knowledge	Bugis	Demonstrated extraction process (*penawar*)
INF-04	Male	65	Ritual assistant	Collection and ritual purification	Bugis	Assisted during plant harvesting and cleansing
INF-05	Female	49	Knowledge custodian	Transmission of healing chants and local terminologies	Bugis	Shared oral narratives and intergenerational teaching contexts

### Sampling and laboratory procedures

Fresh leaves of *F. septica* were collected from five sampling sites within and around Balleanging Village (4°56′ S, 119°40′ E), situated on karstic terrain with altitudes ranging from 70 to 140 m a.s.l. The sites included settlement peripheries, stream banks, and secondary vegetation zones. Each site was geo-referenced using a handheld GPS (Garmin eTrex 32x), and environmental notes (soil type, light exposure, co-occurring species) were recorded.

Leaves collected from the five sampling sites were combined into a composite sample prior to drying and extraction. This composite sampling design was intended to represent the general phytochemical profile of *F. septica* within the study area rather than to compare site-specific variation. The extraction was performed as a single exploratory profiling procedure. Collected leaves were air-dried for seven days (25–30 °C), ground, and extracted with methanol (1:10 w/v) via 72-hour maceration. The extract was filtered and concentrated using a rotary evaporator (40 °C, reduced pressure).

Phytochemical analysis was conducted using a Shimadzu QP2010 Ultra GC–MS system fitted with an Rtx-5MS column (30 m × 0.25 mm × 0.25 μm). The oven temperature was programmed from 60 °C (2-min hold) to 280 °C at 10 °C/min, with helium (1 mL/min) as the carrier gas, injector temperature 250 °C, and ionization voltage 70 eV. Compounds were identified via NIST20M1 library, considering similarity indices ≥ 90% as reliable. Identified peaks were cross-referenced with literature on *Ficus* species’ bioactive compounds.

### Procedure and research design

This study employed a mixed qualitative–analytical design that integrated ethnographic fieldwork, ethnobotanical documentation, and phytochemical profiling to investigate the cultural and biochemical significance of *F. septica* in the karst ecosystems of South Sulawesi. The overall research design followed a biocultural framework, connecting three interrelated stages: [1] ethnographic documentation of traditional knowledge and practices; [2] ethnobotanical and ecological observation of *F. septica* within its natural habitat; and [3] laboratory-based phytochemical analysis to identify bioactive compounds corresponding to local healing concepts.

- Stage I – Ethnographic and ethnobotanical documentation

Fieldwork was conducted between June and September 2025 in Balleanging Village, Pangkajene and Islands Regency, South Sulawesi. Ethnographic methods included semi-structured interviews, participant observation, and free-listing to capture local terminologies, beliefs, and practices related to *F. septica*. Five key informants, comprising traditional healers (*sanro*), elderly knowledge holders, and community herbalists were purposively selected from a population of approximately 12 households.

Data collection focused on local identification, nomenclature, and classification of *F. septica* within Bugis–Makassar cosmology; traditional preparation methods, dosage systems, and treatment categories (e.g., fever, inflammation, spiritual imbalance); perceived efficacy and moral-symbolic interpretations of healing rituals; and observations of plant harvesting, ritual cleansing, and usage contexts.

All interviews were conducted in Bugis languages and later translated into Indonesian and English for analysis. Field notes, photographs, and audio recordings were used to document emic perspectives and embodied practices. Ethical protocols were followed in accordance with the Guidelines for Ethical Research in Ethnobiology [37], with prior informed consent and acknowledgment of community intellectual rights.

- Stage II – Ecological and sampling procedures

Parallel to ethnographic inquiry, ecological data were collected to document the spatial distribution and habitat characteristics of *F. septica*. Five sampling sites were selected within and around Balleanging Village, each representing distinct microhabitats such as settlement edges, stream banks, and limestone slopes. Environmental variables—including soil condition, light exposure, and co-occurring vegetation—were recorded to contextualize the species’ adaptive ecology in karst environments. Fresh leaves were harvested following local protocols of respect and offering, reflecting the community’s cosmological ethic toward plant spirits. These protocols included verbal permission addressed to the plant before cutting, the avoidance of harvesting during certain times considered spiritually inappropriate, and the offering of small symbolic gestures such as sprinkling water or expressing gratitude to acknowledge the plant’s *sumange’* (vital force). Samples were air-dried at 25–30 °C for seven days, pulverized, and extracted with analytical-grade methanol (1:10 w/v) through maceration for 72 h. The filtrate was concentrated using a rotary evaporator at 40 °C under reduced pressure to obtain crude extract.

- Stage III – Phytochemical analysis

Phytochemical profiling of *F. septica* leaf extract was performed using GC–MS with a Shimadzu QP2010 Ultra system equipped with an Rtx-5MS column (30 m × 0.25 mm × 0.25 μm). The temperature program was set from 60 °C (2-min hold) to 280 °C at a rate of 10 °C/min, with helium as the carrier gas (1 mL/min flow rate). Injector temperature was maintained at 250 °C and ionization at 70 eV. Compounds were identified based on spectral matching with the NIST20M1 library, using only similarity indices ≥ 90% as reliable. Identified peaks were then cross-referenced with existing literature on *Ficus* species to verify pharmacological relevance. The GC–MS analysis was conducted as qualitative phytochemical profiling to identify major constituents. This study did not aim to quantify site-level variation or perform replicate-based metabolomic comparison.

- Stage IV – Data triangulation and analytical integration

**Fig. 2:**
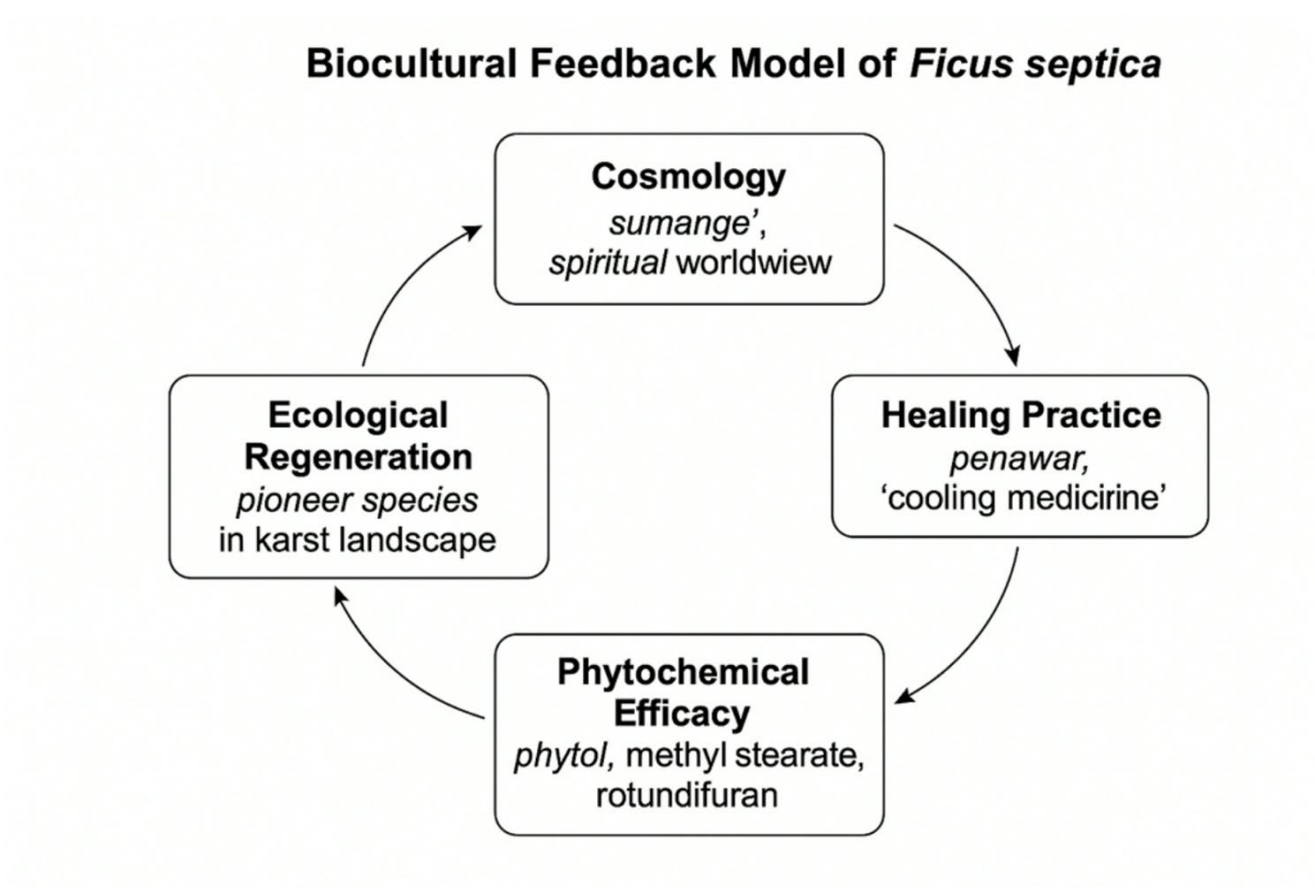
Biocultural Feedback Model of *Ficus septica*. The diagram illustrates the cyclical relationship between cosmology, healing practice, phytochemical efficacy, and ecological regeneration within the Bugis–Makassar worldview. Local cosmology, grounded in the concepts of *sumange’* (vital life force) and *assitinajang* (cosmic balance), informs healing practices in which *F. septica* is used as *penawar*—a “cooling medicine” to restore harmony between body, spirit, and environment. The pharmacological activities of its principal bioactive compounds—such as phytol, methyl stearate, and rotundifuran—provide empirical validation of these cultural interpretations. Ecologically, *F. septica* functions as a pioneer species that stabilizes karst soils and supports frugivorous fauna, reinforcing a feedback loop in which cultural knowledge, biochemical function, and ecosystem processes sustain one another

The analytical phase integrated qualitative ethnographic data with quantitative chemical results through interpretive triangulation. Local ethnomedical classifications such as *panasa’* (heat-related disorders), *penawar* (cooling medicine), and *assitinajang* (cosmic balance) were compared to the pharmacological properties of compounds detected in GC–MS profiling (e.g., phytol, methyl stearate, rotundifuran, myristic acid esters). This cross-analysis enabled the formulation of the Biocultural Feedback Model of *F. septica* (Fig. 2), illustrating how indigenous cosmology, healing practice, biochemical efficacy, and ecological regeneration form a feedback loop that sustains both cultural and environmental resilience.

### Data analysis and interpretation

Data analysis in this study employed an interpretive approach integrating ethnographic narratives, ethnobotanical observations, and phytochemical evidence to understand how *F. septica* operates as both a medicinal and ecological agent in the karst landscapes of South Sulawesi. All interviews and field notes were transcribed and subjected to thematic content analysis. Coding was conducted inductively, allowing recurrent patterns related to plant identity, therapeutic classification, ritual practice, and cosmological interpretation to emerge from the data rather than being imposed a priori. Codes were iteratively refined through discussion among research team members to ensure interpretive consistency. Thematic analysis revealed that participants consistently described *F. septica* as a “cooling” (*penawar*) plant used to counteract bodily or spiritual “heat” (*panasa’*), and as a manifestation of *sumange’*, the life force that connects humans, plants, and the spiritual realm. These cultural categories were then interpreted within the framework of moral ecology, emphasizing that healing practices are not merely physiological but also moral acts of restoring *assitinajang*—a state of cosmic balance between human and environmental domains.

Ethnobotanical data derived from interviews and free-listing were used to assess the cultural salience of *F. septica* across informants. All participants identified it as a principal medicinal species, indicating recurrent identification across informants. Observations of harvesting and preparation practices further revealed that the plant is embedded within ritual ethics, where offerings and verbal acknowledgment accompany each act of collection, reinforcing the notion that healing entails reciprocal relations with non-human beings.

Phytochemical analysis complemented these ethnographic findings by providing biochemical evidence for the plant’s perceived properties. GC–MS profiling detected fifty-four compounds, with major constituents including myristic acid vinyl ester (9.85%), phytol (7.43%), 6-octadecenoic acid methyl ester (3.10%), methyl stearate (0.87%), and rotundifuran (0.27%). These compounds have been reported in previous pharmacological studies to exhibit anti-inflammatory, antioxidant, and antimicrobial activities. The correspondence between these documented bioactivities and local “cooling” classifications is interpreted here as a conceptual alignment rather than as direct experimental validation within this study. In local terms, the pharmacological actions of these molecules correspond to the plant’s capacity to “reduce heat,” “soothe internal imbalance,” and “revive vitality,” reflecting an epistemological overlap between indigenous knowledge and biomedical understanding.

By triangulating ethnographic interpretation, botanical observation, and laboratory data, this study suggests that the conceptual coherence between *sumange’*, *penawar*, and the bioactivity of *F. septica* constitutes an integrated biocultural system. The plant’s healing efficacy, cosmological symbolism, and ecological resilience converge to form what can be described as a “biocultural feedback” process, in which human–plant relationships simultaneously sustain health, moral order, and ecosystem regeneration. In this way, the empirical and cosmological dimensions of *F. septica* mutually reinforce one another, providing both scientific validation and cultural insight into how local communities in South Sulawesi enact conservation through their everyday healing practices. The integrative interpretation presented here is analytical and inferential, aiming to explore correspondence between cultural categories and phytochemical findings, rather than to establish causal biomedical equivalence.

## Results

### Phytochemical profiling of *F. septica* leaf extract

**Fig. 3:**
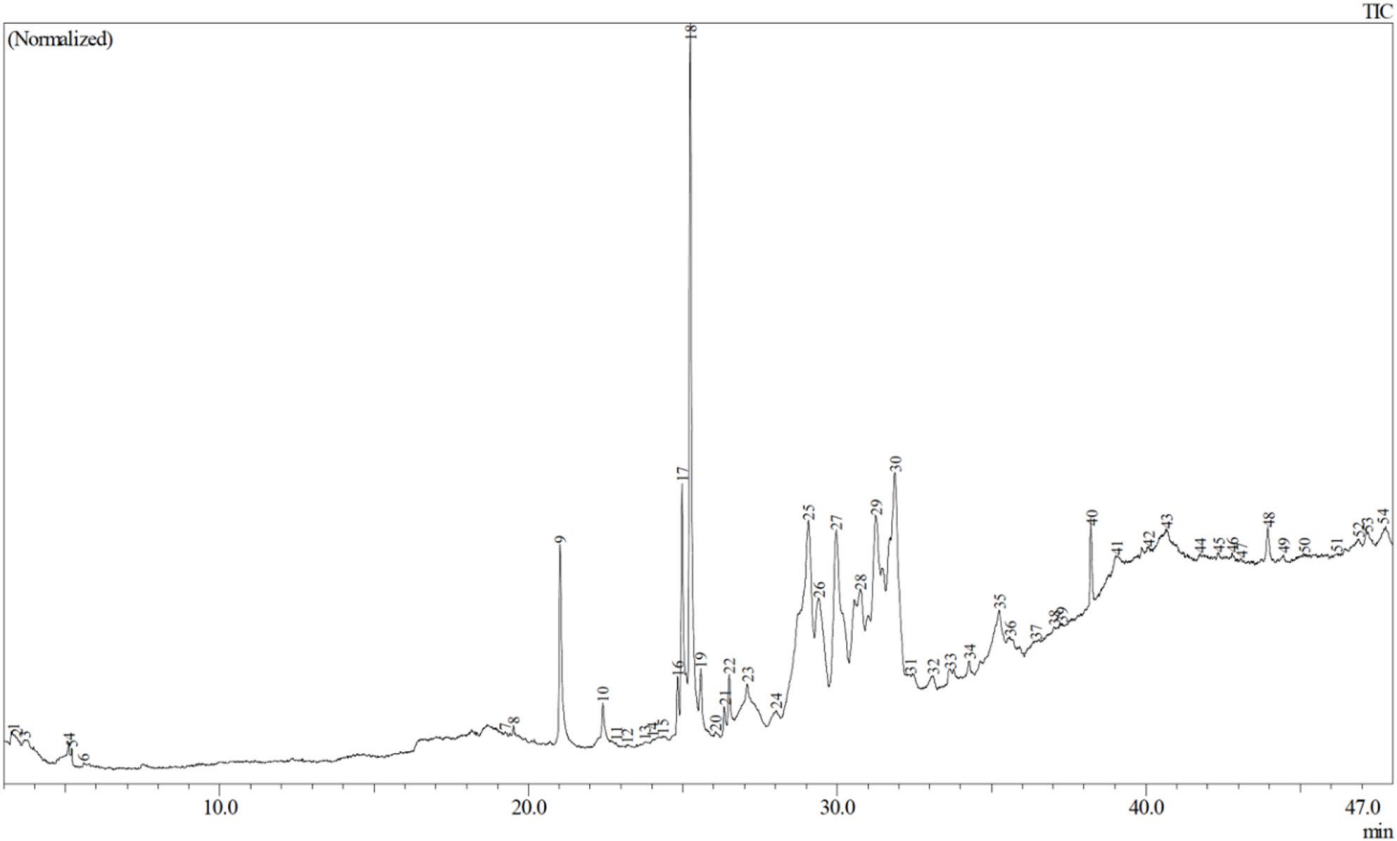
Total Ion Chromatogram (TIC) of *F. septica* showing 54 peaks

GC–MS analysis of the methanolic extract of *F. septica* leaves identified fifty-four compounds with diverse pharmacological and ecological functions. The total ion chromatogram (TIC) showed prominent peaks between 24.8 and 43.9 min, corresponding to fatty acid esters, terpenoids, and phenolic derivatives (Fig. 3).

The most abundant constituents were myristic acid vinyl ester (9.85%), phytol (7.43%), 5,5-diethyltridecane (8.56%), 1-(cyclopropylcarbonyl)-3-piperidinamine, N-trimethylacetyl- (8.30%), Spiro[4.5]decan-7-one (5.26%), and 2,6,10,14,18-pentamethyl-2,6,10,14,18-eicosapentaene (3.01%). These compounds collectively represent over one-third of the total extract area^1^, indicating a complex matrix of bioactive metabolites associated with antioxidant, antimicrobial, and anti-inflammatory activities (Table 2).

**Table 2:** Major bioactive compounds identified in *F. septica* leaf extract (methanol) using GC–MS

No	Retention time (min)	Compound name	Area (%)	Known biological activities	Reported pharmacological activity and cultural interpretation
9	21.03	Hexadecanoic acid, methyl ester (methyl palmitate)	2.15	Antioxidant, anti-inflammatory	Cooling agent (*penawar*)
17	24.98	6-Octadecenoic acid, methyl ester (Z)	3.10	Antioxidant, immune modulator	Restores bodily balance
18	25.25	Phytol	7.43	Antimicrobial, anti-inflammatory	“Cooling” and cleansing agent
19	25.58	Methyl stearate	0.87	Anti-inflammatory, emollient	Wound healing, balance of “heat”
25	29.08	Myristic acid vinyl ester	9.85	Skin-protective, cell-repairing	Calming and protective plant spirit
34	34.28	Rotundifuran	0.27	Cytotoxic, antioxidant	Restores *sumange’* (vital energy)
40	38.23	2,6,10,14,18-Pentamethyl-2,6,10,14,18-eicosapentaene	3.01	Antibacterial, anti-tumor	Strengthening of life force
43	40.68	1-(Cyclopropylcarbonyl)-3-piperidinamine, N-trimethylacetyl-	8.30	Cytoprotective, antibacterial	Purification of internal “heat”
41	39.04	Spiro[4.5]decan-7-one, 1,8-dimethyl-8,9-epoxy-4-isopropyl-	5.26	Analgesic, anti-inflammatory	Spiritual cooling and pain relief

The relative peak areas reported here reflect chromatographic abundance within the composite extract and should not be interpreted as precise quantitative concentration measurements across populations. Several of the identified compounds have been reported in previous studies to exhibit anti-inflammatory and antioxidant properties. The observed correspondence between these documented bioactivities and local “cooling” classifications is interpreted here as conceptual alignment rather than as direct causal confirmation.

### Local uses and healing practices

In the karst village of Balleanging, *F. septica* represents one of the most widely acknowledged medicinal plants in everyday life. All interviewed knowledge holders consistently identified this species as a primary remedy for ailments associated with *“panasa’”* (internal heat), headache, fever, and swelling, which are perceived not merely as physiological disturbances but as indicators of cosmic disequilibrium. Within the local medical framework, *awar-awar* functions as a *penawar*—a neutralizing or cooling agent that absorbs excessive *sumange’* (vital heat) and restores equilibrium between the individual and the surrounding environment.

“When the head throbs and the body feels hot, the *tobo-tobo* leaf is placed on the forehead. It absorbs the body’s heat and soothes the *sumange’* (vital spirit). When the leaf dries, it is replaced with a new one—as if exchanging heat for coolness.” *(Elder and healer*,* Balleanging*,* 2025).*

The preparation process, while straightforward, is highly structured: fresh leaves are harvested directly from plants located near household drainage channels or rocky slopes, slightly crushed to release the latex, and then applied to the forehead, temples, or chest. Some practitioners opt to wrap the leaves in a clean piece of white cloth and moisten it prior to application, reflecting an ethic of purity *(mappasitinaja)* and intention *(pakkatenning sumange’).* This healing practice thus encompasses both biophysical and moral dimensions: alleviating fever while reaffirming the respectful relationship between humans and plants.

During field observations, researchers noted that leaf harvesting was seldom performed casually. Before picking, the healer often recited a brief invocation—seeking permission from the plant and acknowledging its *sumange’*. These ritualized actions, consistently observed in Balleanging and neighboring karst villages, exemplify what [38] describes as a “sacred ecology,” where ecological practice and spiritual discipline intersect in daily resource use. The act of plucking a leaf becomes both medicinal and devotional.

This moral-ecological dimension is also encapsulated in the aphorism frequently cited by community members:

*“Apa yang tumbuh di sekitar rumah adalah obat dari alam”* — what grows around the home is medicine from nature.

The phrase functions as both a moral code and an ecological heuristic, positing that health is intrinsically linked to place: the closer the plant, the more potent its healing properties, as its *sumange’* resonates with the human inhabiting the same environment. This logic is consistent with ethnophysiological observations in other regions of Indonesia, where proximity and familiarity determine the efficacy of medicine [16]. Consequently, *F. septica* serves not only as an herbal remedy but also as a node of relational reciprocity, connecting body, home garden, and cosmic order.

Ethnographic interviews further elucidate that the distinction between physical and spiritual illness is fluid. Fevers are occasionally attributed to the disturbance of *sumange’*—an energetic imbalance resulting from fatigue, emotional tension, or environmental disrespect. Healing, therefore, necessitates both topical treatment and moral realignment. The healer’s role is to “cool” not only the body but also the social and spiritual atmosphere surrounding the patient. Through *awar-awar*, illness becomes a site where cosmology is enacted: plants act as mediators reconnecting human life to the natural and divine realms *(botting langi’).*

These observations reflect the broader cosmological framework of Bugis–Makassar biocultural knowledge, wherein plants, humans, and deities are interconnected in a dynamic chain of vitality. In Sureq Galigo, it is recounted that when *Batara Guru* descended to the middle world, he disseminated the seeds of plants and animals as the culmination of creation. This mythic act positions plants like *tobo-tobo* not merely as biological entities but as cosmological agents entrusted with maintaining equilibrium *(assitinajang)* in both human and environmental domains.

Field data corroborate the continued relevance of this cosmology in contemporary practice. Numerous elders referred to *tobo-tobo* as *“tanaman paccelling”*—the “plant of cooling,” symbolizing composure, restraint, and patience—qualities integral to Bugis ethical life. Within the bissu ritual lexicon, the plant is occasionally invoked in purification ceremonies, reinforcing its symbolic association with restoration and serenity. Comparative ethnobotanical research across Southeast Asia yields parallel insights. *F. septica* has been documented for its use in treating fever, edema, and skin inflammation [18, 39–43]. observed that across twelve ethnomedicinal contexts, the species is consistently associated with “cooling” or “balancing” remedies, reflecting a cross-cultural continuity in its perceived thermal and moral properties. Pharmacological assays provide further support: purified extracts of *F. septica* exhibit anti-inflammatory and COX-2 inhibitory activity (IC₅₀ ≈ 32 µg/mL) ([44], while its alkaloids—*ficuseptine*, *antofine*, and *septicine*—demonstrate antimicrobial and cytotoxic effects [45].

When examined through a biocultural lens, these findings elucidate how empirical efficacy and cosmological logic mutually reinforce each other. The concept of *panasa’* (heat) and *penawar* (cooling) parallels biomedical notions of thermoregulation and anti-inflammatory action. However, within local epistemology, these are not opposing categories but complementary forces whose balance constitutes health. Healing thus becomes an act of ecological alignment, wherein chemical and spiritual harmonies coalesce. In summary, the local healing practice surrounding *F. septica* in Balleanging embodies a synthesis of pharmacological function, cosmological significance, and ecological ethics. The plant serves simultaneously as a medicine, a teacher of moderation, and a living bridge connecting human well-being to the vitality of the karst landscape.

### Cosmological and symbolic dimensions

This section draws primarily on ethnographic interviews conducted in 2025, supplemented by references to classical Bugis texts and anthropological literature to contextualize local interpretations. In the Bugis–Makassar worldview, the ontology of plants transcends mere biological utility. *Tobo-tobo* or *daussalo* (*F. septica)* is perceived as a living intermediary among the realms of the human *(ale kawa)*, the natural *(ale lino)*, and the celestial *(botting langi’).* Within this tripartite cosmology, each organism is endowed with *sumange’*, the vital force that animates existence. The potency of a plant’s *sumange’* not only determines its medicinal efficacy but also its moral status as a being that co-participates in maintaining cosmic order *(assitinajang)*.

“The *tobo-tobo* leaf has a soul. If we take it carelessly, it becomes angry; but if we ask for permission, it will help us.” *Healer*,* Balleanging*,* field notes*,* 2025).*

This ethnographic insight elucidates a metaphysics of reciprocity, wherein the healer’s act of seeking permission prior to harvesting is not indicative of superstition but rather represents an ethical contract—a form of relational accountability akin to what [46] describes as animic ontology, where humans and nonhumans share interiority but differ in physicality. Within this ontology, illness is perceived as a disruption in the moral-ecological fabric that binds humans and the environment; thus, the application of *F. septica* serves to restore not only the patient’s health but also cosmic equilibrium.

The cosmological narrative further reinforces this worldview. Several informants referred to cosmological narratives associated with Batara Guru, describing the dispersal of plants as part of the primordial ordering of the world (field interviews, 2025). This motif resonates with accounts found in the classical Bugis epic *Sureq Galigo*, where vegetation is portrayed as a divine element completing cosmic creation [47]. Consequently, *tobo-tobo* and other *tanaman paccelling* (“cooling plants”) are perceived as manifestations of divine benevolence—agents ensuring balance among the elements of fire *(api’)*, water *(banyu)*, wind *(angin)*, and earth *(tana).* In this schema, fever *(panasa’)* is seen as excessive heat that disrupts *sumange’*, while *awar-awar* functions as a cooling mediator restoring equilibrium.

The plant’s symbolic “cooling” quality resonates with moral virtues esteemed in Bugis ethics: patience *(malempu)*, composure *(sabbara)*, and restraint *(mappasitinajang)*. As a healer explained, “A person who often uses *tobo-tobo* leaves has a cool heart and does not get angry easily.” This association positions *F. septica* as both a botanical and ethical remedy—curing the body and tempering the soul. Similar associations between plant “coolness” and moral equilibrium have been documented in other ethnobotanies [9–50], reinforcing the profound cultural semiotics of thermal balance across the region.

From a phenomenological perspective, the utilization of *tobo-tobo* embodies what [51] describes as the dwelling perspective—knowledge that arises from lived interaction with the landscape. The karst environment, characterized by its sparse vegetation and harsh limestone surfaces, is perceived as spiritually significant yet fragile; plants that manage to grow there are seen as embodying resilience and divine favor. Consequently, the ability of *awar-awar* to thrive on barren rocks symbolizes the persistence of life and moral order in marginal ecologies—a potent metaphor within local cosmology.

During field interviews, one ritual practitioner described the use of *tobo-tobo* leaves in purification rites (field notes, 2025). In healing and blessing rituals, *tobo-tobo* leaves are sometimes employed as offerings or purification media, alongside sacred water and rice flour. According to one *bissu*, the plant’s *sumange’ “menyejukkan dunia dan hati”—*cooling both the world and the heart. Through such ritual deployment, *F. septica* transcends its biological identity, functioning as a symbolic bridge between the visible and invisible worlds.

This cosmological role aligns with contemporary scholarship on biocultural ethics and ecological personhood, which frames indigenous environmental practices as moral enactments of interspecies kinship [36, 52]. Within this framework, *tobo-tobo* is not merely an ethnopharmacological specimen but a biocultural signifier—its healing acts reaffirm the ontological unity of beings that inhabit the karst ecosystem. This unity sustains the notion that health is harmony, and illness is dissonance between moral, ecological, and spiritual forces.

Interestingly, this indigenous semiotic system converges with scientific recognition of *Ficus* species as ecological keystones that sustain tropical food webs [53, 54]. In local cosmology, this ecological indispensability translates into spiritual indispensability—*awar-awar* becomes *panasa’ na sumange’* (“the coolness of vitality”), a metaphor for resilience. The convergence of cosmological meaning and ecological function exemplifies the biocultural coherence that underlies traditional environmental knowledge systems [55].

Thus, the cosmological and symbolic dimensions of *F. septica* reveal a sophisticated epistemology that integrates ontology, ethics, and ecology. The plant’s perceived *sumange’* and “cooling” character not only elucidate its medicinal role but also articulate a worldview where healing is an act of cosmological rebalancing. Such understanding challenges Western separations between medicine, spirituality, and environment—offering instead a holistic model where every act of healing is simultaneously a reaffirmation of life’s interconnected web.

### Ecological role in karst systems

**Fig. 4:**
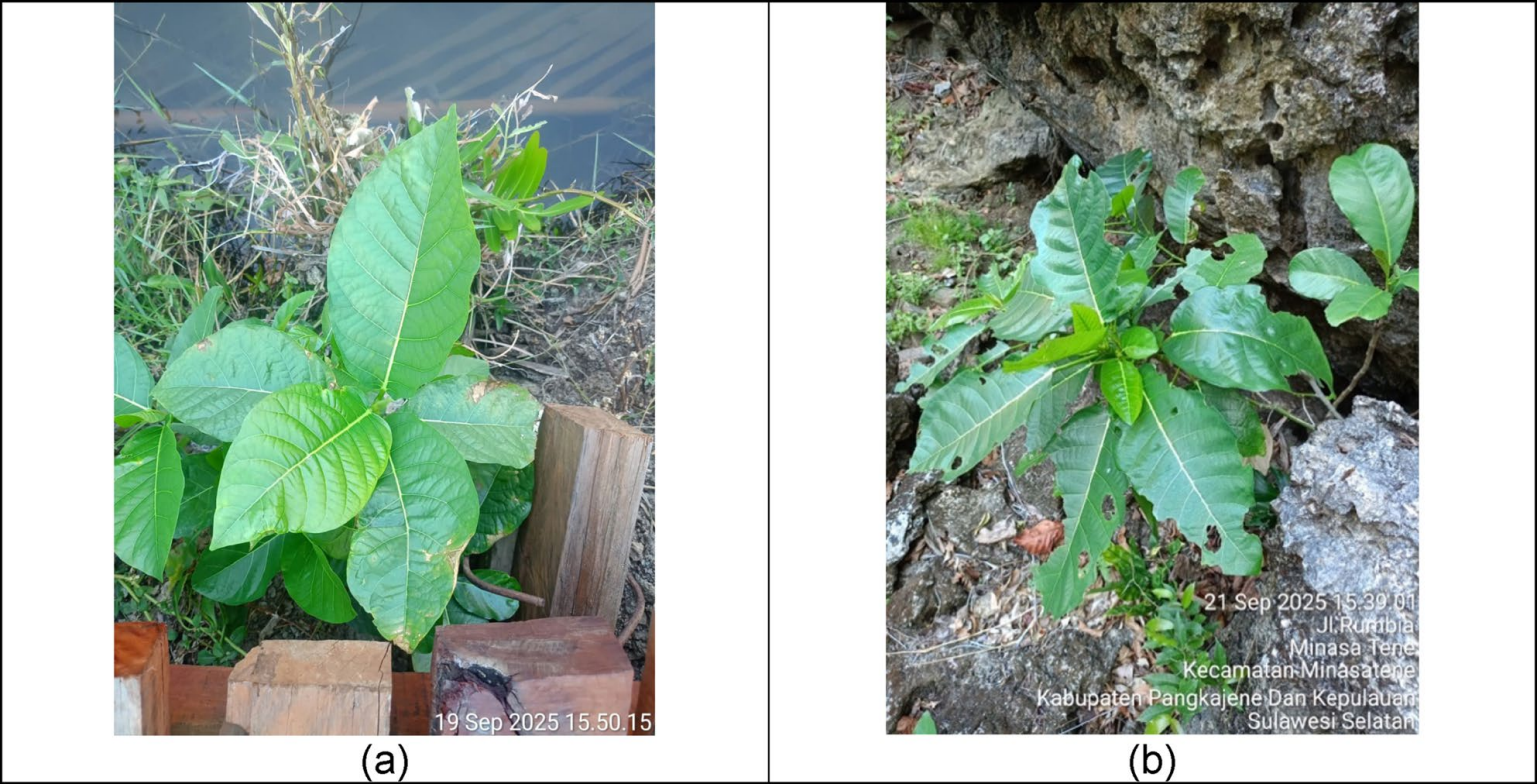
*F. septica* (*tobo-tobo/awar-awar*): (**a**) leaves commonly used in traditional healing to treat fever and heat imbalance; (**b**) wild plant growing among limestone rocks, indicating its resilience and ecological role in karst landscapes

The ecological presence of *F. septica* within the Balleanging karst landscape complements its medicinal and symbolic roles. In the limestone terrain of South Sulawesi—characterized by thin soils and exposed rock surfaces—the species was repeatedly observed growing in rocky crevices, settlement margins, and disturbed slopes (Fig. 4). These field observations indicate that *F. septica* exhibits traits consistent with pioneer species commonly associated with early-stage vegetation establishment in karst environments.

Its roots were observed penetrating limestone fissures and accumulating organic matter around mature individuals. Although soil stabilization and erosion rates were not quantitatively measured, the recurrent presence of *F. septica* in exposed microhabitats suggests a potential contribution to localized substrate stabilization. These interpretations are observational and supported by broader ecological literature on Ficus species in tropical systems.

These ecological attributes are consistent with broader studies of *Ficus* ecology across Southeast Asia. *Ficus* species are widely recognized as keystone taxa in tropical ecosystems due to their role in structuring plant–animal interactions and facilitating secondary succession [53, 54]. As noted by [56], *Ficus* species sustain frugivore populations during periods of food scarcity, effectively acting as “nutritional bridges” that stabilize local biodiversity. In karst regions where canopy resources are sparse and soils are poor, *F. septica* thus provides both structural and trophic support to the ecosystem.

In Balleanging, community members often refer to *awar-awar* as *tanaman yang menghijaukan batu*—“the plant that greens the stones.” This metaphor encapsulates a local ecological understanding that recognizes *F. septica* as a regenerative force within degraded habitats. Its persistence on infertile limestone substrates is perceived as a sign of ecological blessing, reinforcing the cultural belief that certain plants are divinely positioned to restore balance between human activity and nature’s resilience. This vernacular ecological perception aligns with scientific accounts of *F. septica’s* physiological tolerance to drought and nutrient-poor conditions, attributed to its efficient water-use strategy and latex-mediated defense system [20, 57].

The plant’s small, pear-shaped figs mature asynchronously throughout the year, attracting fruit bats *(Pteropodidae)*, bulbuls *(Pycnonotidae)*, and barbets (*Megalaimidae)*—key frugivores in karst environments. During fieldwork, opportunistic observations documented instances of fruit bats (*Cynopterus brachyotis*) feeding on *F. septica* fruits at forest edges. These observations, together with established literature on Ficus–frugivore interactions in Southeast Asia, suggest that the species likely participates in local seed dispersal networks, although frugivore visitation frequency was not systematically quantified in this study [58–61]. These frugivores disperse seeds across fragmented patches, contributing to natural regeneration corridors that extend from homesteads to secondary forests.

From an ecological network perspective, the relationship between *F. septica* and frugivores constitutes a core mutualistic link that enhances karst biodiversity. Its continuous fruiting ensures year-round food availability, a trait characteristic of *Ficus* keystone species [62]. In turn, frugivores facilitate seed dispersal over topographically complex terrains, maintaining genetic connectivity among isolated vegetation clusters—a critical factor in the fragmented limestone ecosystems of Maros–Pangkep.

At the local scale, mature individuals were observed to provide canopy cover and litter accumulation that may influence microclimatic conditions. However, the extent of these effects was not quantitatively assessed. The dense canopy of mature specimens reduces soil temperature and enhances organic litter accumulation, thereby creating favorable microsites for the establishment of seedlings of other species, such as *Diospyros maritima*,* Premna serratifolia*, and *Artocarpus elasticus*. This ecological niche promotes secondary succession, a phenomenon also documented in the karst systems of Luzon [56]. These observations suggest that F. septica exhibits traits consistent with pioneer species in karst habitats, contributing to microhabitat formation and facilitating subsequent vegetation establishment. While the present study did not quantify soil stabilization rates or successional dynamics, field observations indicate that the species participates in early-stage regeneration processes in limestone environments.

In Balleanging, local cosmology acknowledges this ecological role in spiritual terms. Villagers perceive the plant’s resilience as a manifestation of *sumange’ tanah*—the “vital spirit of the land”—exemplified by species capable of thriving on stone while providing shade, fruit, and medicinal resources. Such interpretations align with the biocultural resilience framework proposed by [36], wherein ecological adaptability is intertwined with cultural valuation. The persistence of *F. septica* on marginal soils reflects the community’s own endurance, both symbolically and materially rooted in the karst’s precarious yet life-sustaining environment.

In summary, Within the scope of this study, F. septica can be interpreted as part of a localized biocultural dynamic linking healing practice, ecological presence, and cultural meaning in karst landscapes. These interpretations should be understood as context-specific and observational rather than experimentally verified ecosystem-level assessments.

## Discussion

The findings of this study demonstrate that the Bugis–Makassar classification of *F. septica* as a “cooling” (*penawar*) medicine reflects a coherent biocultural logic that aligns with its molecular pharmacology. Within the local medical framework, the concept of *panasa’* (heat) refers not only to fever but also to emotional agitation, exhaustion, and disturbances of *sumange’*—the vital force linking the body, landscape, and spiritual domains. The application of *F. septica* leaves to neutralize this excess heat is therefore part of a broader cosmological ethics of restoring *assitinajang*—cosmic balance. Ethnographic analysis indicates that this thermal logic is not metaphorical but stems from accumulated empirical observations embedded in ritual practice. This aligns with [63], who argue that indigenous medical taxonomies often emerge from systematic experiential experimentation rather than symbolic reasoning alone.

Comparable patterns have been documented in other socio-ecological contexts where traditional medical knowledge contributes to ecological resilience. Studies among Amazonian communities, for example, show that medicinal plant classifications are embedded in cosmological systems that regulate harvesting practices and forest regeneration [64–69]. In the Pacific Islands, ritualized plant use has similarly been shown to function as informal conservation governance by restricting extraction and reinforcing intergenerational transmission of ecological knowledge [36]. Across Southeast Asia, humoral classifications linking “heat” and “coolness” to plant properties also shape selective harvesting and habitat stewardship. In this broader comparative perspective, the Bugis–Makassar case illustrates how therapeutic classification, moral ecology, and ecological presence converge to reinforce both species persistence and cultural continuity. Rather than being incidental to conservation, healing practices themselves may operate as everyday mechanisms of ecosystem resilience.

The GC–MS analysis offers supportive phytochemical context for these cultural interpretations, although no bioactivity assays were conducted in the present study. Compounds such as phytol, methyl palmitate, methyl stearate, and rotundifuran—identified in the present study—have well-documented anti-inflammatory, antioxidant, and cytoprotective effects [70–72]. Previous pharmacological studies have reported that compounds such as phytol and methyl esters may act on inflammatory pathways, including NF-κB and COX-2. In the present study, these mechanisms are referenced as literature-based evidence that provides contextual support for local therapeutic classifications, rather than as experimentally verified effects of the analyzed extract. This biochemical–cultural alignment supports [73] argument that medicinal plant knowledge often represents a form of ecological pattern recognition. The local concept of “cooling” therefore encodes observable anti-inflammatory outcomes—translated not into molecular language but into moral–ecological terms tied to the integrity of the person and the land. This culturally embedded thermal classification can be further understood through the concept of “mental herbals” proposed by [74], which emphasizes that local plant knowledge is structured through context-sensitive cognitive maps shaped by landscape, memory, and lived experience. In this perspective, categories such as *panasa’* and *penawar* are not symbolic metaphors but elements of a situated classificatory system grounded in everyday interaction with plants and environments. The Bugis–Makassar notion of “cooling” thus reflects a cognitive-ecological ordering of medicinal efficacy rather than a purely cosmological abstraction.

Beyond its medicinal role, *F. septica* operates as a biocultural keystone species whose ecological functions mirror its cultural significance. Its ability to colonize limestone crevices, stabilize shallow soils, and maintain year-round fruiting makes it central to karst ecosystem dynamics. Ficus species more broadly are recognized globally as ecological keystones for their role in supporting frugivore networks and facilitating successional recovery [53, 54]. These ecological attributes of *F. septica* directly correspond to local perceptions that the plant “greens the stones” and embodies the regenerative force of the landscape. The plant’s persistence in degraded karst terrain reinforces cultural narratives that divine resilience and moral order are embedded in species capable of thriving under environmental stress. Such convergence between ecological performance and cosmological meaning echoes [36], who contend that biocultural keystone species sustain both biodiversity and cultural identity.

This embedded relationship between plant, place, and meaning resonates with [75] concept of the “herbal landscape,” which frames medicinal knowledge as inseparable from the ecological and spatial contexts in which it is practiced. In Balleanging, the karst terrain is not merely a physical backdrop but an active agent in shaping ethnomedical understanding. The visibility of *F. septica* on limestone slopes, its resilience in thin soils, and its proximity to households reinforce its cultural status as a cooling and restorative plant. The karst landscape thus functions as a knowledge-producing environment, where ecological conditions continuously inform medicinal classification and symbolic interpretation.

Interpreted through a biocultural conservation lens, the reciprocal relationship between local knowledge and ecological function has direct implications for conservation strategies in karst landscapes. The belief that *F. septica* possesses *sumange’*—a vital force deserving respect—creates behavioral norms that restrict destructive harvesting and encourage care in collection. These cultural protocols constitute a form of practical conservation consistent with [52] model of biocultural ethics, wherein moral relations between humans and nonhumans shape ecological stewardship. In Balleanging, ritualized practices of asking permission before harvest, avoiding unnecessary cutting, and selecting only fresh leaves embody a culturally embedded governance system over plant use. Such practices align with contemporary conservation frameworks advocating community-based management that integrates local cosmologies with ecological knowledge.

The integration of local cosmological principles into formal conservation planning offers significant opportunities for strengthening karst restoration strategies. Recognizing *F. septica* as a culturally protected species, rather than only a biological resource, could be institutionalized through participatory conservation agreements involving village authorities, customary councils, and research agencies such as BRIN. Embedding the concept of *assitinajang* (balance) into environmental education and local regulations may further cultivate behavioral norms that promote sustainable resource use. Similar approaches have shown success in other biocultural landscapes, where linking moral concepts to ecological indicators enhances community commitment to conservation [17, 50].

Moreover, *F. septica* provides a practical bridge between ethnomedicine and habitat restoration. As a pioneer species, it naturally rehabilitates degraded limestone surfaces, allowing it to function simultaneously as medicinal infrastructure and ecological engineer. Integrating *F. septica* propagation into karst rehabilitation programs could therefore advance both public health and ecosystem resilience—an approach aligned with the global movement toward nature-based solutions that incorporate cultural values [56]. Strengthening local cultivation, protecting wild stands, and monitoring frugivore interactions would form a holistic strategy grounded in both ecological processes and cultural legitimacy.

Overall, the convergence of indigenous thermodynamic logic, phytochemical evidence, and ecological function underscores that *F. septica* is not simply a medicinal resource but an axis of biocultural continuity. Its role in healing the body, stabilizing the landscape, and reaffirming cosmological order makes it an exemplary model of how plants mediate relationships between humans and fragile ecosystems. For conservation policy, this means that protecting the species and its habitat is inseparable from safeguarding the epistemologies and moral systems that give it meaning. Effective karst conservation must therefore integrate TEK, community institutions, and ecological science—acknowledging that biocultural keystone species like *F. septica* sustain not only biodiversity but also the cultural resilience necessary for long-term stewardship of vulnerable landscapes.

## Conclusions

This study suggest that *F. septica* may function as a biocultural keystone species whose significance extends beyond its medicinal value to encompass ecological resilience and cosmological meaning within the Bugis–Makassar worldview. The convergence between indigenous thermodynamic classifications, particularly the concepts of *panasa’* (heat), *penawar* (cooling medicine), *sumange’* (vital force), and *assitinajang* (balance), and the plant’s reported anti-inflammatory and antioxidant properties documented in previous pharmacological studies indicates a meaningful alignment between local epistemologies and phytochemical findings, rather than a direct causal equivalence. The presence of phytol, methyl esters, rotundifuran, and other bioactive compounds provides molecular evidence that aligns with indigenous interpretations of healing, demonstrating that local medical epistemologies encapsulate adaptive strategies refined through generations of ecological engagement.

As a pioneer species thriving in the harsh conditions of the Maros–Pangkep karst, *F. septica* contributes to soil stabilization, microhabitat formation, and frugivore networks, thereby reinforcing its ecological relevance within this specific landscape context. Its cultural salience and ecological performance can be interpreted as forming a biocultural feedback dynamic, in which healing practices, cosmological ethics, and ecological processes interact. This interpretation should be understood as heuristic and context-specific, given the limited number of informants and the single-site scope of the study. Maintaining biocultural diversity therefore involves not only conserving species, but also safeguarding the knowledge systems and moral frameworks that shape human–environment relationships.

For conservation planning, these findings suggest the potential value of integrating TEK into karst management policies. Recognizing *F. septica* as a culturally protected species may enhance participatory conservation initiatives, particularly when ecological restoration efforts are grounded in community values and ritual ethics. Embedding the principle of *assitinajang* into education and local governance could contribute to fostering environmentally responsible practices, although further comparative research across sites and methodological approaches—including expanded bioactivity testing—would be necessary to substantiate broader generalizations. In this sense, the conservation of *F. septica* offers a promising case for exploring biocultural strategies that align ecological rehabilitation with cultural resilience. Sustaining the species, its habitats, and the associated knowledge systems remains an important avenue for strengthening both biodiversity and culturally grounded environmental stewardship.

This study has several contextual limitations. The ethnographic component involved a limited number of key informants (*n* = 5), deliberately selected for their recognized expertise in traditional healing practices. This focused sampling strategy was intended to facilitate in-depth exploration of specialized knowledge and cosmological interpretations rather than to achieve representational breadth. However, such an approach does not encompass the full diversity of perspectives that may exist within the broader Bugis–Makassar community. In addition, the research was conducted in a single study site—Balleanging Village—intentionally chosen for its location within a karst ecosystem in order to examine plant–landscape relationships in a limestone context. While this site-specific focus enabled detailed contextual analysis, the findings should be understood as situated within this particular socio-ecological setting and may not be directly generalizable to non-karst environments or other regional contexts.

## Supplementary Information

Below is the link to the electronic supplementary material.

Supplementary Material 1

Supplementary Material 2

## Data Availability

All datasets generated or analyzed during this study are available in the BRIN Dataverse Repository at the following persistent link: [https://hdl.handle.net/20.500.12690/RIN/7ZFF5X](https:/hdl.handle.net/20.500.12690/RIN/7ZFF5X) . The repository includes anonymized ethnographic transcripts, field photographs, GC–MS chromatogram files, and supporting metadata under controlled access. Data can be obtained from the corresponding author upon reasonable request and in accordance with BRIN data-sharing policies.

## Footnotes

1 The associations presented in the final column represent interpretive correspondences between published pharmacological findings and locally documented healing classifications. These do not constitute experimental validation within the present study.
