# Supplementary material for: From healing landscapes to biocultural conservation: the role of Ficus septica in karst ecosystems of South Sulawesi, Indonesia
Source: J Ethnobiol Ethnomed. 2026 Apr 4;22:46. doi: 10.1186/s13002-026-00896-3 (PMC13169875; doi:10.1186/s13002-026-00896-3)
Supplement: Supplementary file 1 — Supplementary Material 1 [file 13002_2026_896_MOESM1_ESM.pdf]

DATA REPORT GCMS-QP2010 ULTRA SHIMADZU

Sample Information

Analyzed by	: Admin
Analyzed	: 23/09/2025 5:41:50 PM
Sample Type	: Unknown
Level #	: 1
Sample Name	: Ekstrak Daun Awar Awar
Sample ID	: Ekstrak Daun Awar Awar
IS Amount	: [1]=1
Sample Amount	: 1

Chromatogram Ekstrak Daun Awar Awar C:\GCMSsolution\Data\Project1\Ekstrak Daun Awar Awart.QGD

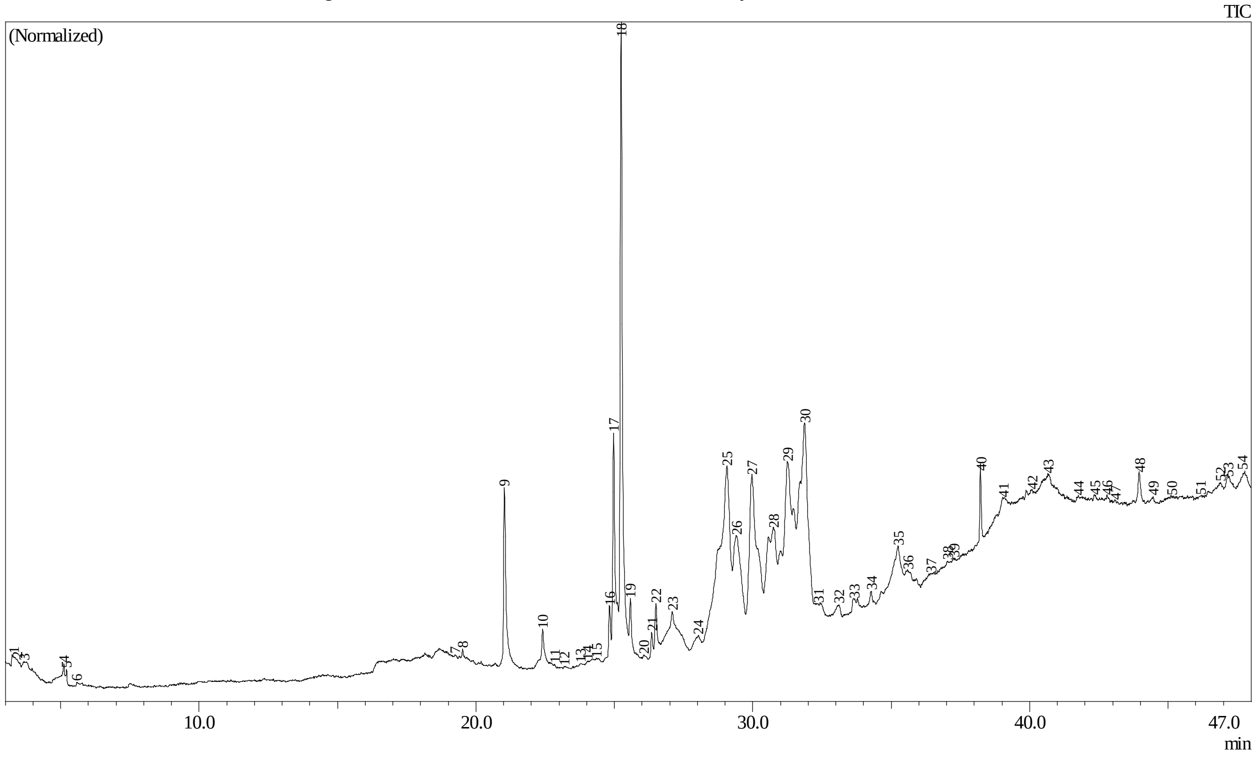

Peak Report TIC

Peak#	R.Time	Area	Area%	Name
1	3.246	295328	0.12	2,2-Dimethoxybutane
2	3.425	346611	0.15	2,2-Dimethoxybutane
3	3.690	361962	0.15	2,2-Dimethoxybutane
4	5.121	312135	0.13	3,3-Dimethoxy-2-butanone
5	5.217	138234	0.06	1,3-Dioxolane-4-methanol, 2-ethyl-
6	5.586	163161	0.07	Nonane
7	19.245	54865	0.02	(-)-Neoclovene-(II), dihydro-
8	19.518	137493	0.06	Neophytadiene
9	21.030	5127882	2.15	Hexadecanoic acid, methyl ester
10	22.413	1761368	0.74	Hexadecanoic acid, ethyl ester
11	22.850	267979	0.11	4-Piperidylmethanol, TMS
12	23.200	43999	0.02	Hexadecanoic acid, 15-methyl-, methyl ester
13	23.767	138957	0.06	4-Ethynyl-6-(piperidin-1-yl)-2,1,3-benzoxadiazole
14	24.033	155217	0.07	5-Hydroxymethyl-2-thioxoimidazolidin-4-one
15	24.358	586435	0.25	1-Hydroxy-3-(octanoyloxy)propan-2-yl decanoate
16	24.832	1621713	0.68	Methyl 10-trans,12-cis-octadecadienoate
17	24.979	7382576	3.10	6-Octadecenoic acid, methyl ester, (Z)-
18	25.249	17690791	7.43	Phytol
19	25.582	2075289	0.87	Methyl stearate
20	26.074	186743	0.08	1,2-Epoxy-5,9-cyclododecadiene
21	26.354	564607	0.24	9,12-Octadecadienoic acid, ethyl ester
22	26.505	1773124	0.74	Dichloroacetic acid, tridec-2-ynyl ester
23	27.092	5623307	2.36	Ethyl 14-methyl-hexadecanoate
24	28.038	923580	0.39	Myristic acid vinyl ester
25	29.075	23463978	9.85	Myristic acid vinyl ester
26	29.419	9705658	4.08	Dodecanoic acid, 2,2,3,3,4,4,4-heptafluorobutyl ester

Peak#	R.Time	Area	Area%	Name
27	29.979	15622119	6.56	Myristic acid vinyl ester
28	30.764	11621736	4.88	Dodecanoic acid, ethenyl ester
29	31.275	20376522	8.56	5,5-Diethyltridecane
30	31.883	21362676	8.97	5,5-Diethyltridecane
31	32.367	1251070	0.53	Tetrahydrorhombifoline
32	33.121	865826	0.36	24-Noroleana-3,12-diene
33	33.668	999377	0.42	Benzoic acid, p-(diethylamino)-, 9-borabicyclo[3.3.1]non-9-yl ester
34	34.286	632946	0.27	Rotundifuran
35	35.257	6208903	2.61	Fumaric acid, 2-methylpentyl dec-2-yl ester
36	35.600	2108469	0.89	Undec-10-ynoic acid, heptyl ester
37	36.433	1307980	0.55	Acetic acid, 3-hydroxy-7-isopropenyl-1,4a-dimethyl-2,3,4,4a,5,6,7,8-octahydronaphthal[illegible]
38	37.033	1983934	0.83	Tetracosanoic acid, methyl ester
39	37.267	1087761	0.46	1,4-Benzenedicarboxylic acid, bis(2-ethylhexyl) ester
40	38.236	7169208	3.01	2,6,10,14,18-Pentamethyl-2,6,10,14,18-eicosapentaene
41	39.046	12536533	5.26	Spiro[4.5]decan-7-one, 1,8-dimethyl-8,9-epoxy-4-isopropyl-
42	40.100	13709254	5.76	2-Butyloxycarbonyloxy-1,1,10-trimethyl-6,9-epidioxydecalin
43	40.684	19768151	8.30	1-(Cyclopropylcarbonyl)-3-piperidinamine, N-trimethylacetyl-
44	41.766	5093873	2.14	N-(2-Hydroxyethyl)-4-methylthio-1,2-carbazoledicarboximide
45	42.367	2366956	0.99	4-Ethylbenzoic acid, undecyl ester
46	42.800	3404984	1.43	Cyclohexanol, 3-ethenyl-3-methyl-2-(1-methylethenyl)-6-(1-methylethyl)-, [1R-(1.alpha.
47	43.100	1070459	0.45	Stigmasta-3,5-diene
48	43.970	3557395	1.49	Benzoic acid, 4-propyl-, hexadecyl ester
49	44.461	650694	0.27	Cycloheptane, 4-methylene-1-methyl-2-(2-methyl-1-propen-1-yl)-1-vinyl-
50	45.130	255293	0.11	8,15-Labdanediol
51	46.167	96260	0.04	Eicosapentaenoic Acid, TMS derivative
52	46.902	913793	0.38	Cyclododecanol, 1-(methoxymethyl)-
53	47.171	874161	0.37	26,27-Dinorcholesta-5,22-dien-3-ol, (3.beta.,22E)-
54	47.775	311932	0.13	14-Methyl-hexadecane-1,2-diol, isopropylidene
		238111257	100.00	
