# Supplementary material for: From healing landscapes to biocultural conservation: the role of Ficus septica in karst ecosystems of South Sulawesi, Indonesia
Source: J Ethnobiol Ethnomed. 2026 Apr 4;22:46. doi: 10.1186/s13002-026-00896-3 (PMC13169875; doi:10.1186/s13002-026-00896-3)

Library

<< Target >>
Line#:1 R.Time:3.242(Scan#:30) MassPeaks:468
RawMode:Averaged 3.233-3.250(29-31) BasePeak:87.05(11036)
BG Mode:Calc. from Peak Group 1 - Event 1 Scan

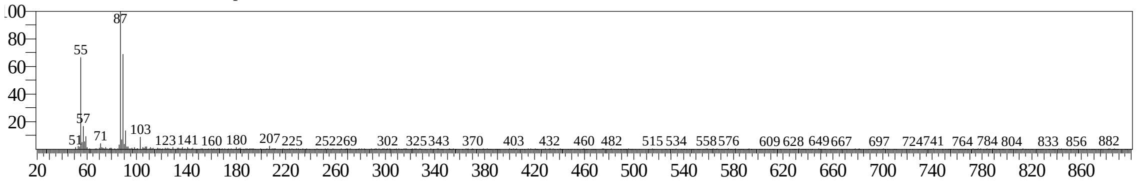

Hit#:1 Entry:5355 Library:NIST20M1.lib
SI:90 Formula:C6H14O2 CAS:3453-99-4 MolWeight:118 RetIndex:685
CompName:2,2-Dimethoxybutane

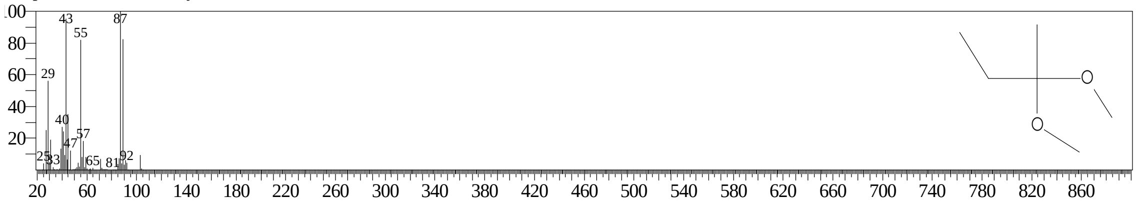

<< Target >>
Line#:2 R.Time:3.425(Scan#:52) MassPeaks:402
RawMode:Averaged 3.417-3.433(51-53) BasePeak:87.05(2924)
BG Mode:Calc. from Peak Group 1 - Event 1 Scan

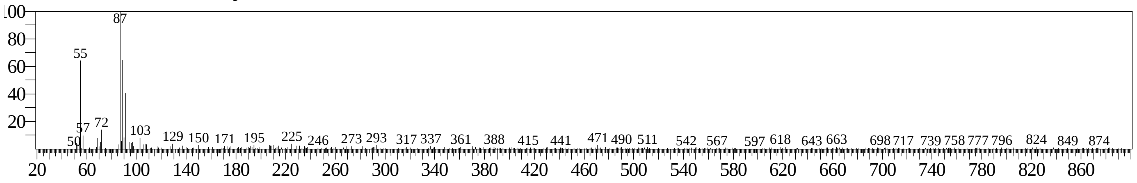

Hit#:1 Entry:5355 Library:NIST20M1.lib
SI:77 Formula:C6H14O2 CAS:3453-99-4 MolWeight:118 RetIndex:685
CompName:2,2-Dimethoxybutane

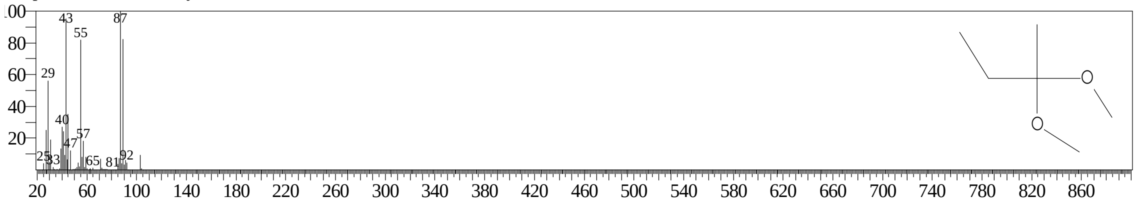

<< Target >>
Line#:3 R.Time:3.692(Scan#:84) MassPeaks:426
RawMode:Averaged 3.683-3.700(83-85) BasePeak:87.05(9367)
BG Mode:Calc. from Peak Group 1 - Event 1 Scan

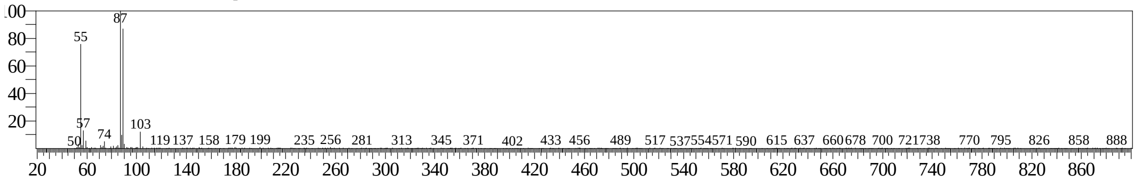

Hit#:1 Entry:5355 Library:NIST20M1.lib
SI:91 Formula:C6H14O2 CAS:3453-99-4 MolWeight:118 RetIndex:685
CompName:2,2-Dimethoxybutane

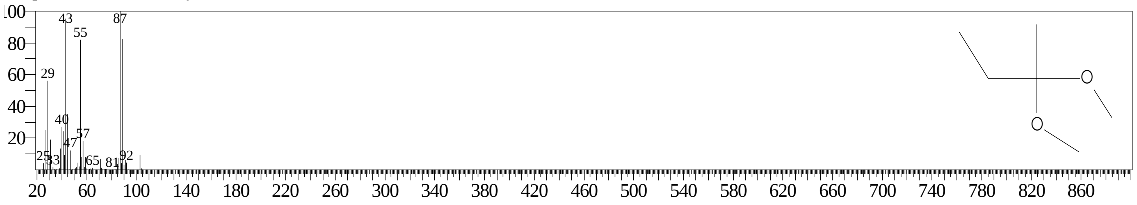

<< Target >>
ine#:4 R.Time:5.125(Scan#:256) MassPeaks:491
awMode:Averaged 5.117-5.133(255-257) BasePeak:89.05(21454)
G Mode:Calc. from Peak Group 1 - Event 1 Scan

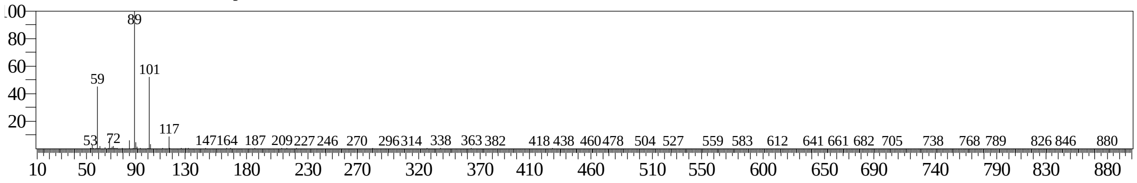

it#:1 Entry:9263 Library:NIST20M1.lib
I:85 Formula:C6H12O3 CAS:21983-72-2 MolWeight:132 RetIndex:821
ompName:3,3-Dimethoxy-2-butanone

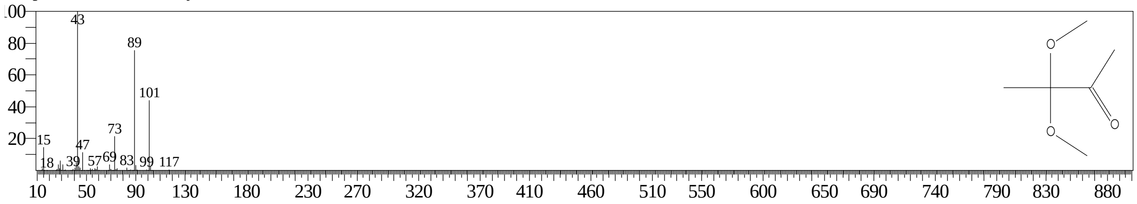

<< Target >>
ine#:5 R.Time:5.217(Scan#:267) MassPeaks:473
awMode:Averaged 5.208-5.225(266-268) BasePeak:103.05(12289)
G Mode:Calc. from Peak Group 1 - Event 1 Scan

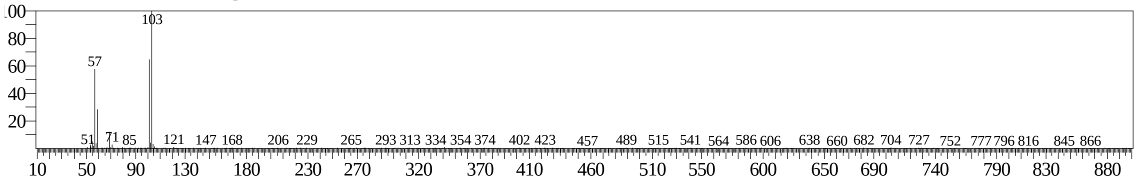

it#:1 Entry:9242 Library:NIST20M1.lib
I:80 Formula:C6H12O3 CAS:53951-44-3 MolWeight:132 RetIndex:1042
ompName:1,3-Dioxolane-4-methanol, 2-ethyl- $$ (2-Ethyl-1,3-dioxolan-4-yl)methanol #

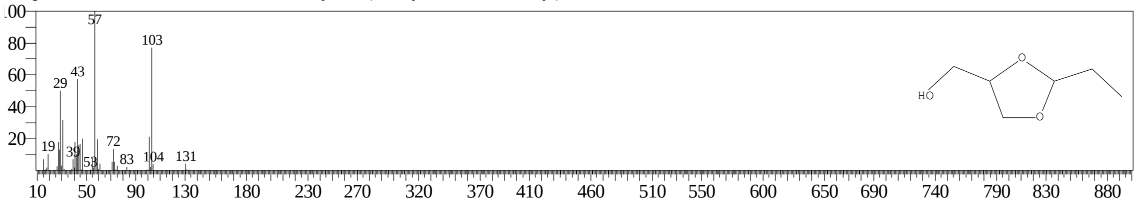

<< Target >>
ine#:6 R.Time:5.583(Scan#:311) MassPeaks:506
awMode:Averaged 5.575-5.592(310-312) BasePeak:57.05(4124)
G Mode:Calc. from Peak Group 1 - Event 1 Scan

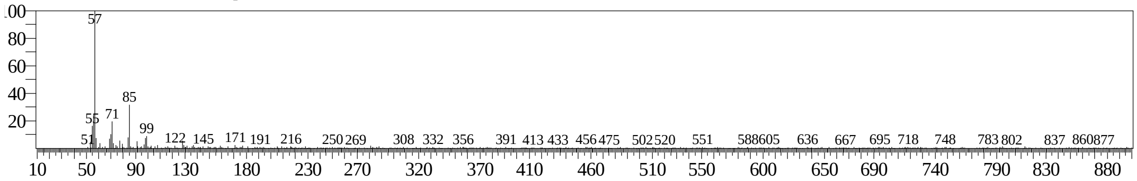

it#:1 Entry:8081 Library:NIST20M1.lib
I:86 Formula:C9H20 CAS:111-84-2 MolWeight:128 RetIndex:900
ompName:Nonane $$ n-Nonane $$ Shellsol 140 $$ n-C9H20 $$ UN 1920

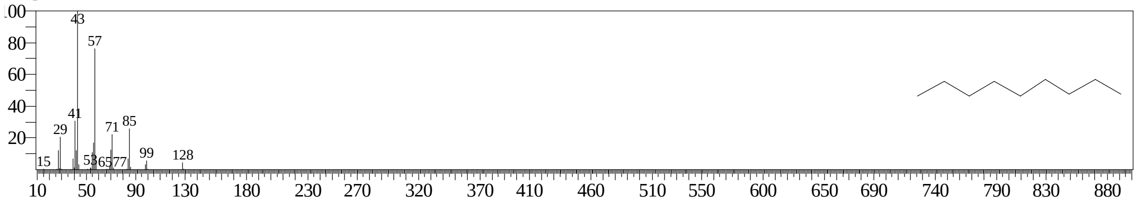

<< Target >>
Line#:7 R.Time:19.242(Scan#:1950) MassPeaks:513
RawMode:Averaged 19.233-19.250(1949-1951) BasePeak:96.05(598)
BG Mode:Calc. from Peak Group 1 - Event 1 Scan

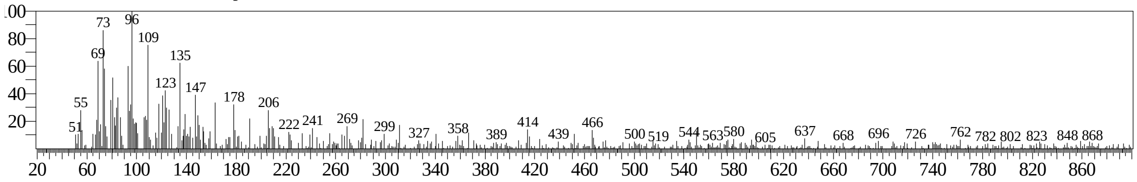

Hit#:1 Entry:59146 Library:NIST20M1.lib
SI:61 Formula:C15H26 CAS:0-00-0 MolWeight:206 RetIndex:1393
CompName:(-)-Neoclovene-(II), dihydro-

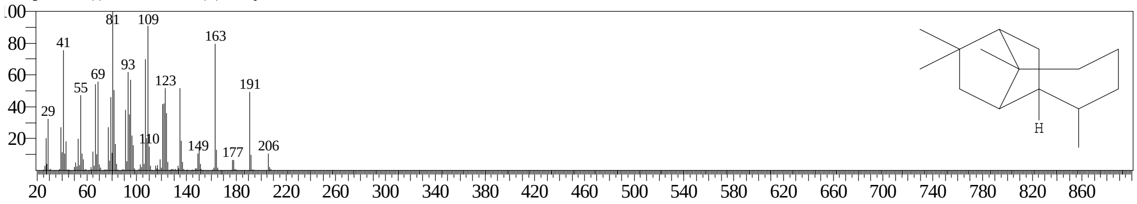

<< Target >>
Line#:8 R.Time:19.517(Scan#:1983) MassPeaks:475
RawMode:Averaged 19.508-19.525(1982-1984) BasePeak:82.05(3361)
BG Mode:Calc. from Peak Group 1 - Event 1 Scan

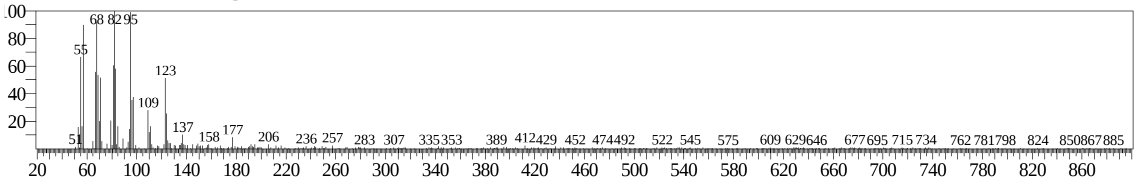

Hit#:1 Entry:137561 Library:NIST20M1.lib
SI:91 Formula:C20H38 CAS:504-96-1 MolWeight:278 RetIndex:1774
CompName:Neophytadiene $$ 7,11,15-Trimethyl-3-methylenehexadec-1-ene $$ 1-Hexadecene, 7,11,15-trimethyl-3-methylene- $$ 1,3-Butadiene, 2-(4,8,12-trim

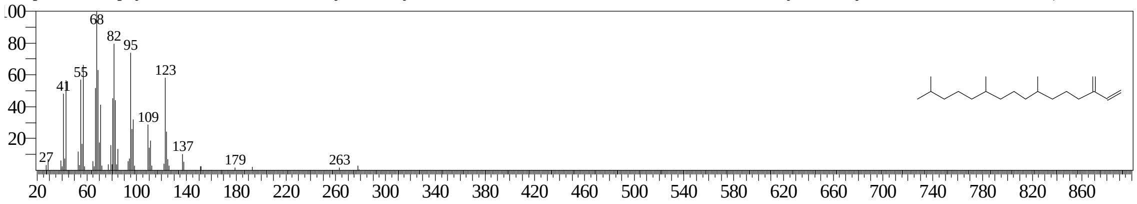

<< Target >>
Line#:9 R.Time:21.033(Scan#:2165) MassPeaks:450
RawMode:Averaged 21.025-21.042(2164-2166) BasePeak:74.05(191403)
BG Mode:Calc. from Peak Group 1 - Event 1 Scan

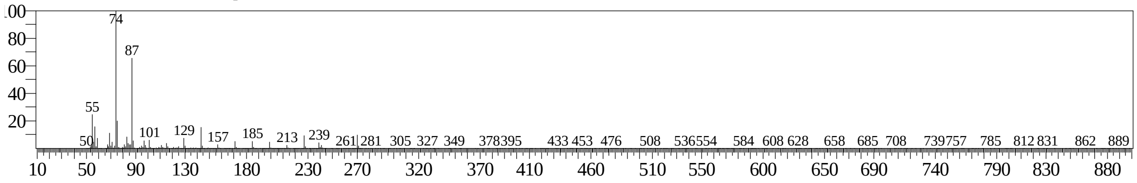

Hit#:1 Entry:128195 Library:NIST20M1.lib
SI:96 Formula:C17H34O2 CAS:112-39-0 MolWeight:270 RetIndex:1878
CompName:Hexadecanoic acid, methyl ester $$ Palmitic acid, methyl ester $$ n-Hexadecanoic acid methyl ester $$ Metholene 2216 $$ Methyl hexadecanoate $

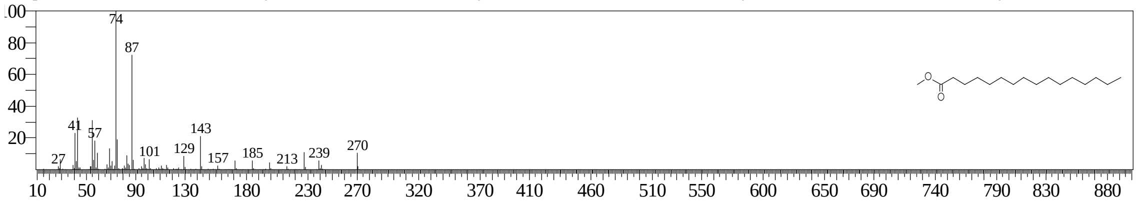

< Target >>
ine#:10 R.Time:22.417(Scan#:2331) MassPeaks:495
awMode:Averaged 22.408-22.425(2330-2332) BasePeak:88.05(31343)
G Mode:Calc. from Peak Group 1 - Event 1 Scan

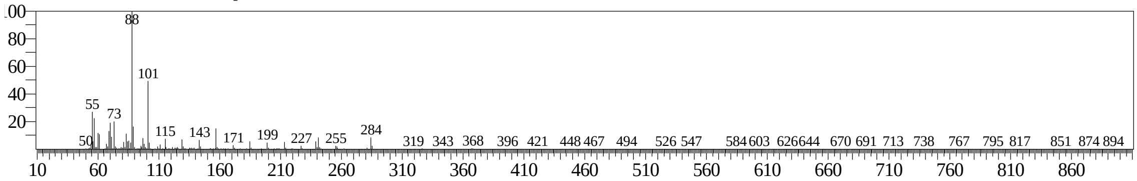

it#:1 Entry:144796 Library:NIST20M1.lib
I:94 Formula:C18H36O2 CAS:628-97-7 MolWeight:284 RetIndex:1978
ompName:Hexadecanoic acid, ethyl ester $$ Palmitic acid, ethyl ester $$ Ethyl hexadecanoate $$ Ethyl palmitate $$ Ethyl n-hexadecanoate

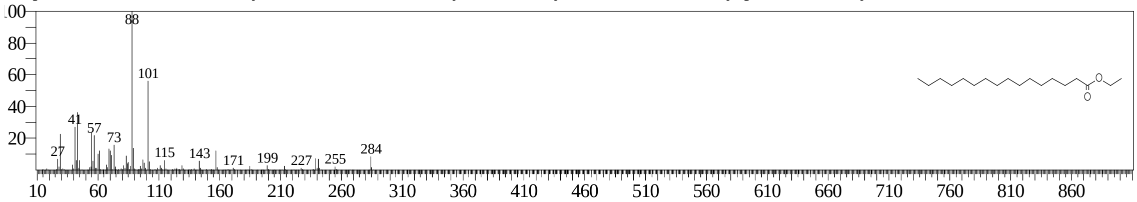

< Target >>
ine#:11 R.Time:22.850(Scan#:2383) MassPeaks:569
awMode:Averaged 22.842-22.858(2382-2384) BasePeak:56.05(422)
G Mode:Calc. from Peak Group 1 - Event 1 Scan

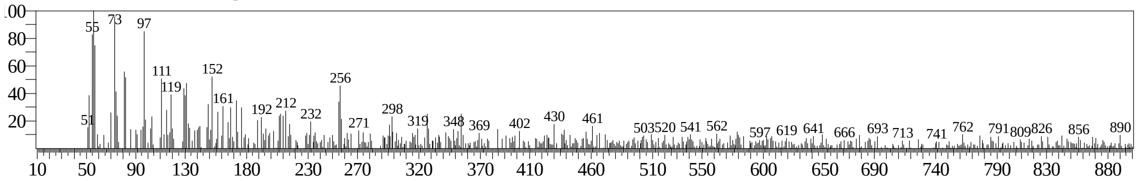

it#:1 Entry:42142 Library:NIST20M1.lib
I:48 Formula:C9H21NOSi CAS:1600978-31-1 MolWeight:187 RetIndex:1210
ompName:4-Piperidylmethanol, TMS $$ Piperidine, 4-[[(trimethylsilyl)oxy]methyl]-

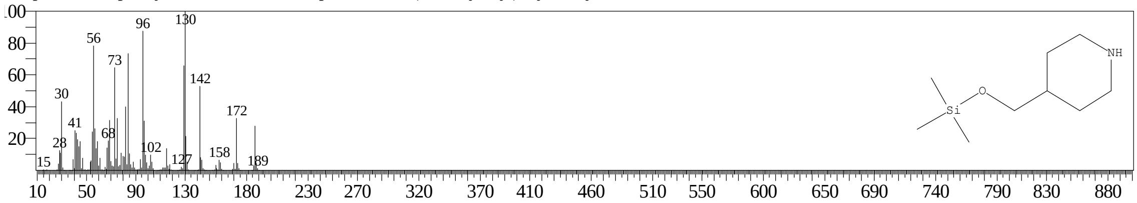

< Target >>
ine#:12 R.Time:23.200(Scan#:2425) MassPeaks:390
awMode:Averaged 23.192-23.208(2424-2426) BasePeak:74.05(1963)
G Mode:Calc. from Peak Group 1 - Event 1 Scan

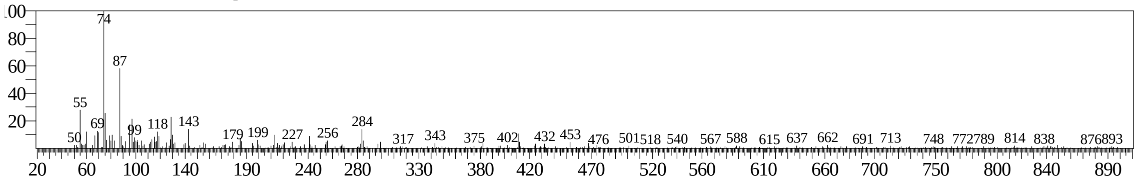

it#:1 Entry:144746 Library:NIST20M1.lib
I:71 Formula:C18H36O2 CAS:6929-04-0 MolWeight:284 RetIndex:1914
ompName:Hexadecanoic acid, 15-methyl-, methyl ester $$ Methyl isoheptadecanoate $$ Methyl 15-methylhexadecanoate

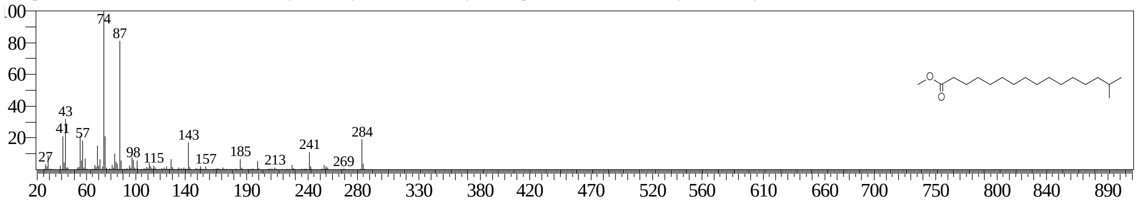

<< Target >>
Line#:13 R.Time:23.767(Scan#:2493) MassPeaks:551
RawMode:Averaged 23.758-23.775(2492-2494) BasePeak:55.05(427)
BG Mode:Calc. from Peak Group 1 - Event 1 Scan

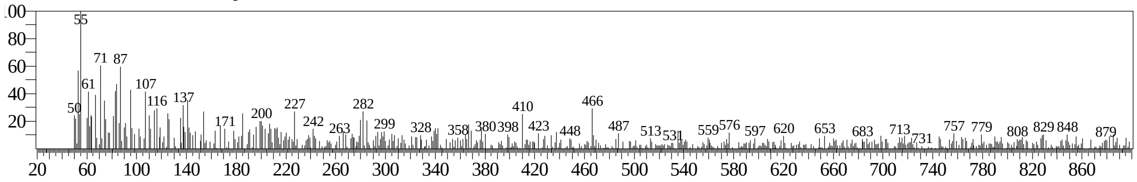

Hit#:1 Entry:80243 Library:NIST20M1.lib
SI:48 Formula:C13H13N3O CAS:0-00-0 MolWeight:227 RetIndex:1920
CompName:4-Ethynyl-6-(piperidin-1-yl)-2,1,3-benzoxadiazole

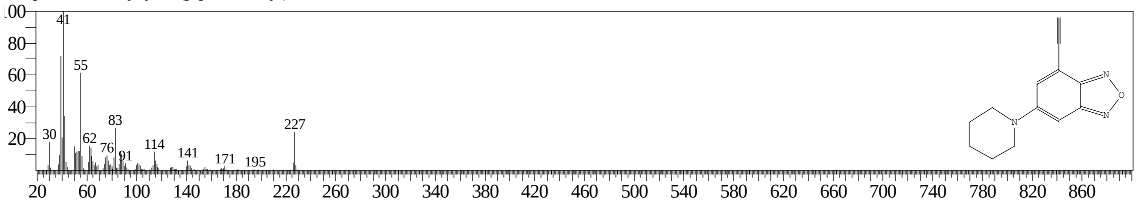

<< Target >>
Line#:14 R.Time:24.033(Scan#:2525) MassPeaks:488
RawMode:Averaged 24.025-24.042(2524-2526) BasePeak:57.05(340)
BG Mode:Calc. from Peak Group 1 - Event 1 Scan

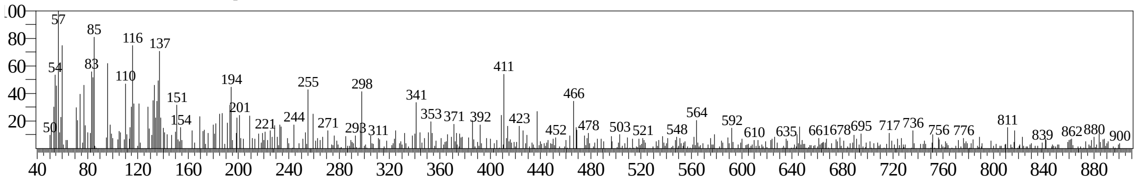

Hit#:1 Entry:14929 Library:NIST20M1.lib
SI:42 Formula:C4H6N2O2S CAS:130629-17-3 MolWeight:146 RetIndex:1521
CompName:5-Hydroxymethyl-2-thioxoimidazolidin-4-one $$ 5-(Hydroxymethyl)-2-thioxo-4-imidazolidinone #

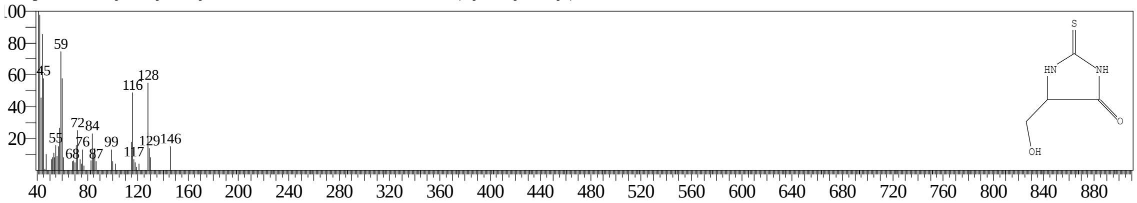

<< Target >>
Line#:15 R.Time:24.358(Scan#:2564) MassPeaks:530
RawMode:Averaged 24.350-24.367(2563-2565) BasePeak:57.05(1034)
BG Mode:Calc. from Peak Group 1 - Event 1 Scan

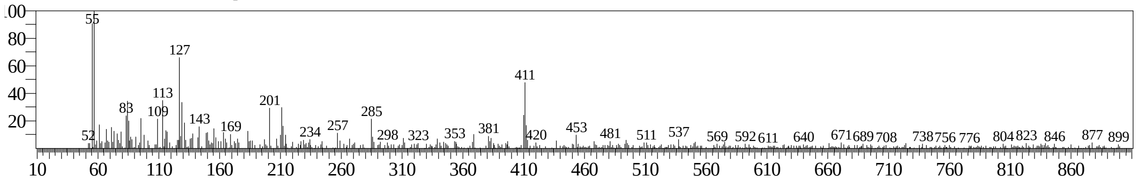

Hit#:1 Entry:240721 Library:NIST20M1.lib
SI:54 Formula:C21H40O5 CAS:177717-46-3 MolWeight:372 RetIndex:2622
CompName:1-Hydroxy-3-(octanoyloxy)propan-2-yl decanoate $$ Decanoic acid, 1-(hydroxymethyl)-2-[(1-oxooctyl)oxy]ethyl ester

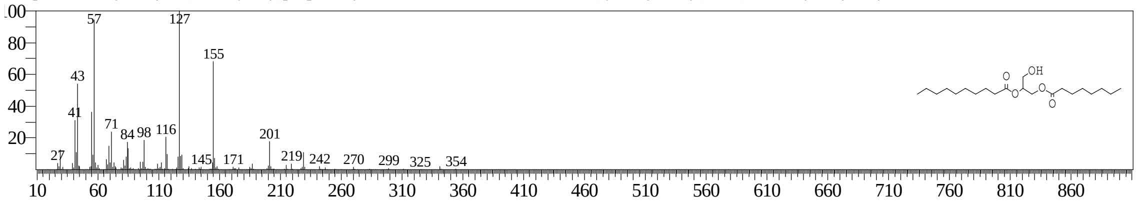

:< Target >>
.ine#:16 R.Time:24.833(Scan#:2621) MassPeaks:522
RawMode:Averaged 24.825-24.842(2620-2622) BasePeak:81.05(15236)
3G Mode:Calc. from Peak Group 1 - Event 1 Scan

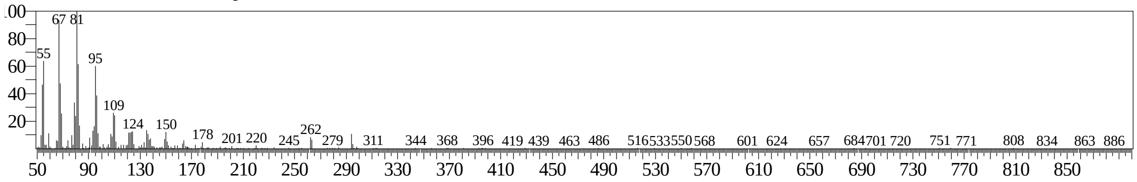

Hit#:1 Entry:156565 Library:NIST20M1.lib
SI:93 Formula:C19H34O2 CAS:0-00-0 MolWeight:294 RetIndex:2093
CompName:Methyl 10-trans,12-cis-octadecadienoate

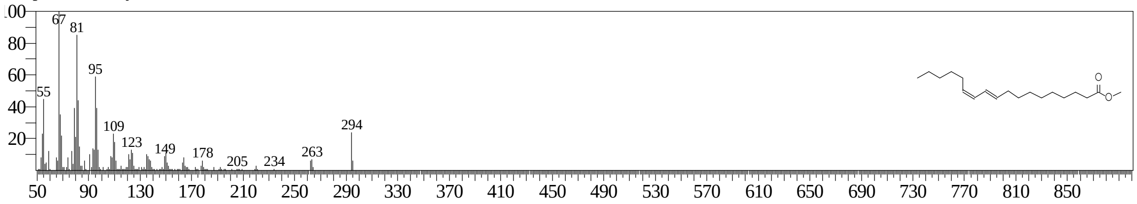

:< Target >>
.ine#:17 R.Time:24.975(Scan#:2638) MassPeaks:550
RawMode:Averaged 24.967-24.983(2637-2639) BasePeak:55.05(68180)
3G Mode:Calc. from Peak Group 1 - Event 1 Scan

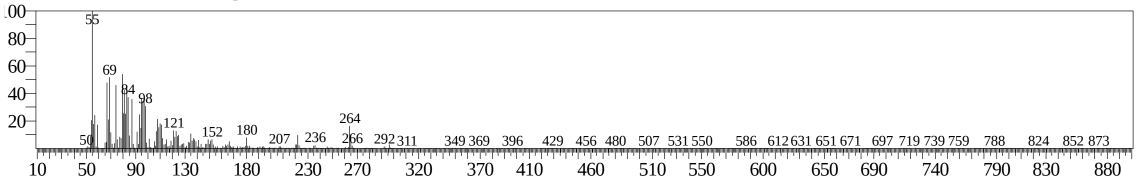

Hit#:1 Entry:158941 Library:NIST20M1.lib
SI:87 Formula:C19H36O2 CAS:2777-58-4 MolWeight:296 RetIndex:2085
CompName:6-Octadecenoic acid, methyl ester, (Z)- $$ Methyl cis-6-octadecenoate $$ Methyl petroselinate $$ Methyl (6Z)-6-octadecenoate # $$ cis-6-Octadec

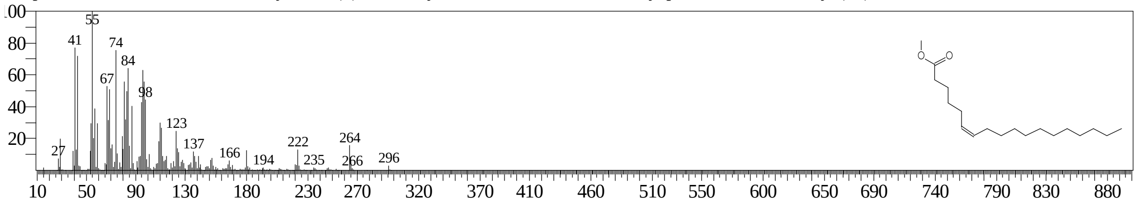

:< Target >>
.ine#:18 R.Time:25.250(Scan#:2671) MassPeaks:563
RawMode:Averaged 25.242-25.258(2670-2672) BasePeak:71.05(524370)
3G Mode:Calc. from Peak Group 1 - Event 1 Scan

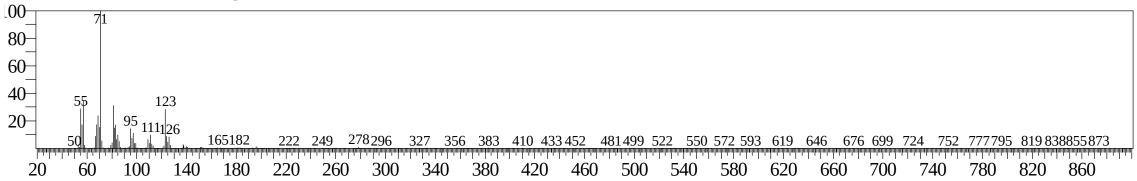

Hit#:1 Entry:159029 Library:NIST20M1.lib
SI:97 Formula:C20H40O CAS:150-86-7 MolWeight:296 RetIndex:2045
CompName:Phytol $$ 2-Hexadecen-1-ol, 3,7,11,15-tetramethyl-, [R-[R*,R*-(E)]]- $$ trans-Phytol $$ 3,7,11,15-Tetramethyl-2-hexadecen-1-ol-, (2E,7R,11R)- $$

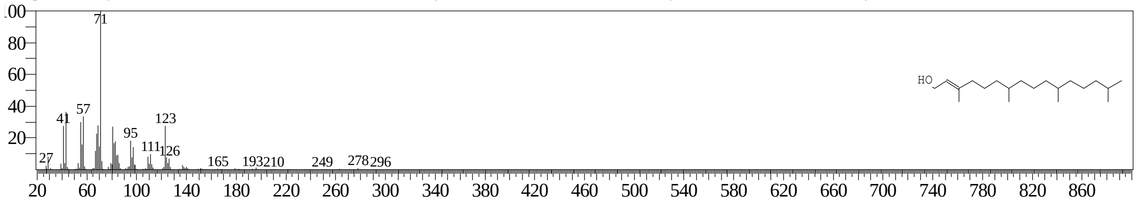

<< Target >>
.ine#:19   R.Time:25.583(Scan#:2711) MassPeaks:471
RawMode:Averaged 25.575-25.592(2710-2712) BasePeak:74.05(44124)
3G Mode:Calc. from Peak Group 1 - Event 1 Scan

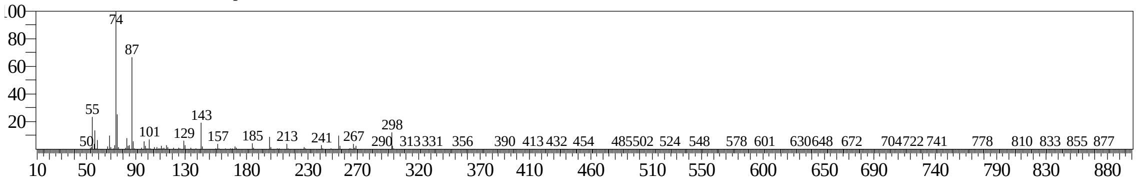

Hit#:1 Entry:161615 Library:NIST20M1.lib
SI:93 Formula:C19H38O2 CAS:112-61-8 MolWeight:298 RetIndex:2077
CompName:Methyl stearate $$ Octadecanoic acid, methyl ester $$ Stearic acid, methyl ester $$ n-Octadecanoic acid, methyl ester $$ Kemester 9718 $$ Methyl r

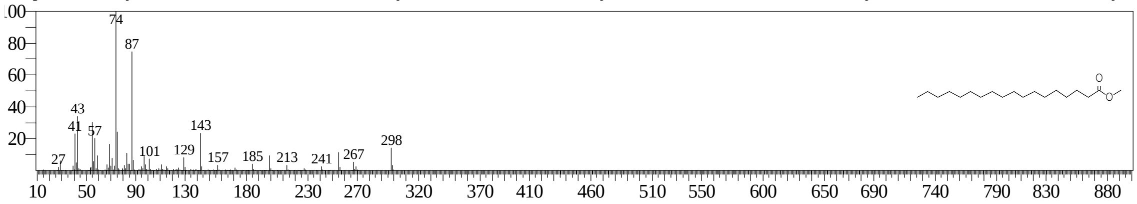

<< Target >>
.ine#:20   R.Time:26.075(Scan#:2770) MassPeaks:442
RawMode:Averaged 26.067-26.083(2769-2771) BasePeak:105.05(1179)
3G Mode:Calc. from Peak Group 1 - Event 1 Scan

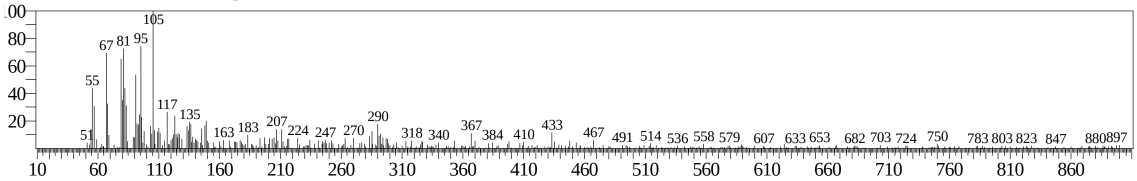

Hit#:1 Entry:35539 Library:NIST20M1.lib
SI:69 Formula:C12H18O CAS:943-93-1 MolWeight:178 RetIndex:1414
CompName:1,2-Epoxy-5,9-cyclododecadiene $$ 13-Oxabicyclo[10.1.0]trideca-4,8-diene

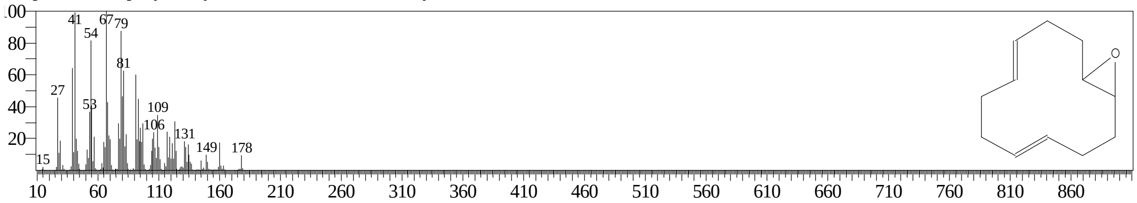

<< Target >>
.ine#:21   R.Time:26.350(Scan#:2803) MassPeaks:461
RawMode:Averaged 26.342-26.358(2802-2804) BasePeak:81.05(8046)
3G Mode:Calc. from Peak Group 1 - Event 1 Scan

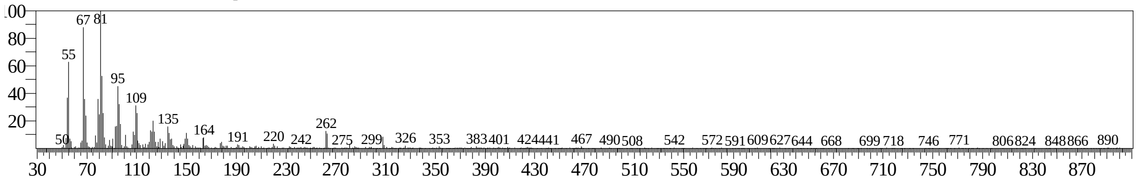

Hit#:1 Entry:173186 Library:NIST20M1.lib
SI:91 Formula:C20H36O2 CAS:7619-08-1 MolWeight:308 RetIndex:2193
CompName:9,12-Octadecadienoic acid, ethyl ester $$ Ethyl-9,12-octadecadienoate $$ Ethyl octadec-9,12-dienoate

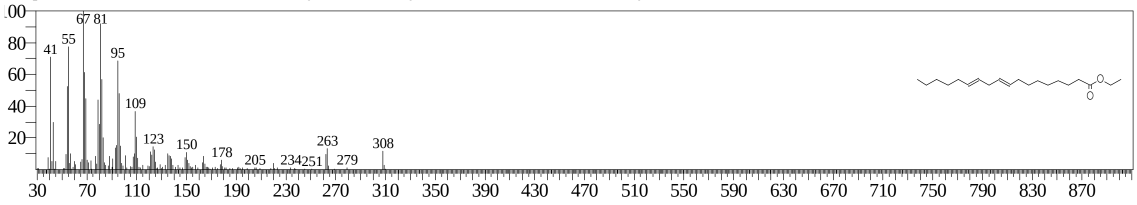

:< Target >>
.ine#:22   R.Time:26.508(Scan#:2822)   MassPeaks:418
RawMode:Averaged 26.500-26.517(2821-2823) BasePeak:79.05(15513)
3G Mode:Calc. from Peak   Group 1 - Event 1   Scan

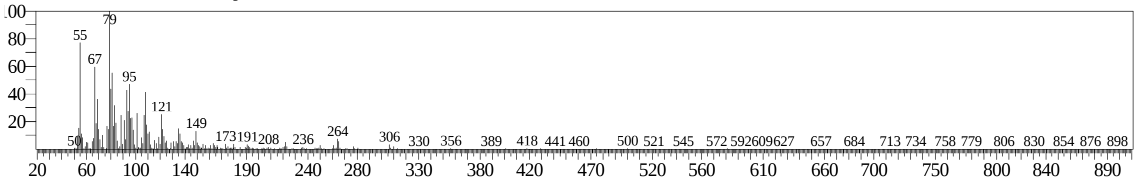

Hit#:1   Entry:170238   Library:NIST20M1.lib
SI:84   Formula:C15H24Cl2O2   CAS:0-00-0   MolWeight:306   RetIndex:2042
CompName:Dichloroacetic acid, tridec-2-ynyl ester

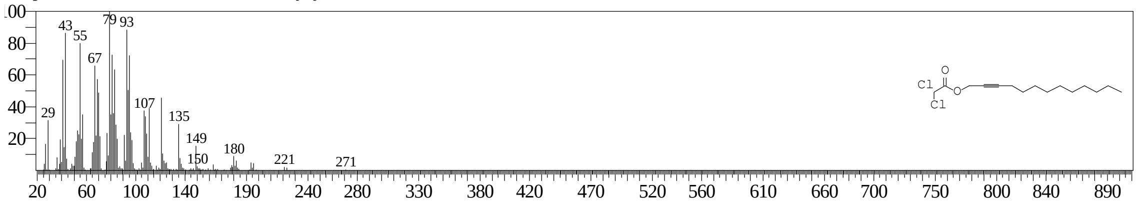

:< Target >>
.ine#:23   R.Time:27.092(Scan#:2892)   MassPeaks:459
RawMode:Averaged 27.083-27.100(2891-2893) BasePeak:57.05(12276)
3G Mode:Calc. from Peak   Group 1 - Event 1   Scan

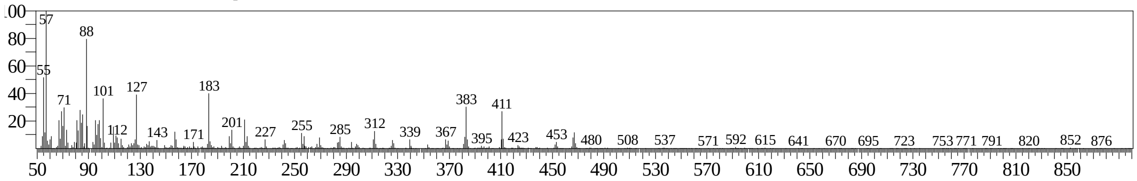

Hit#:1   Entry:161645   Library:NIST20M1.lib
SI:72   Formula:C19H38O2   CAS:0-00-0   MolWeight:298   RetIndex:2013
CompName:Ethyl 14-methyl-hexadecanoate

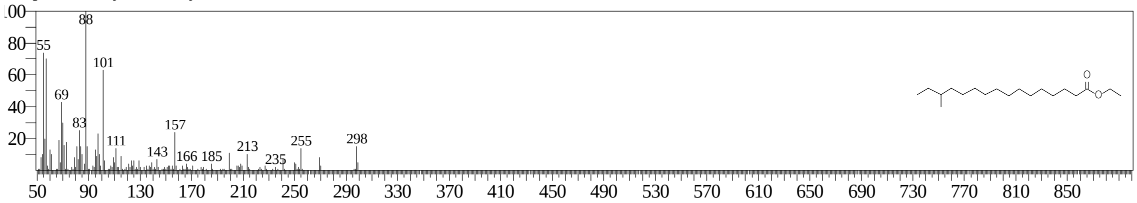

:< Target >>
.ine#:24   R.Time:28.042(Scan#:3006)   MassPeaks:512
RawMode:Averaged 28.033-28.050(3005-3007) BasePeak:57.05(3414)
3G Mode:Calc. from Peak   Group 1 - Event 1   Scan

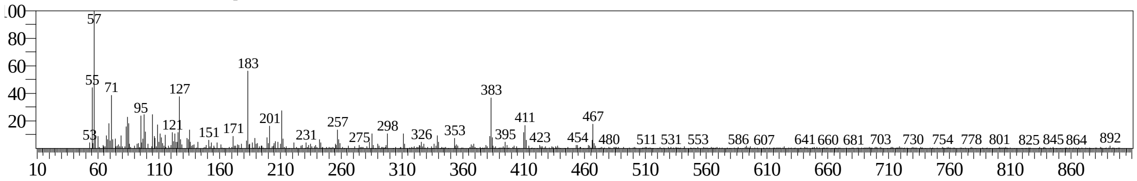

Hit#:1   Entry:109740   Library:NIST20M1.lib
SI:60   Formula:C16H30O2   CAS:5809-91-6   MolWeight:254   RetIndex:1769
CompName:Myristic acid vinyl ester $$ Vinyl myristate #

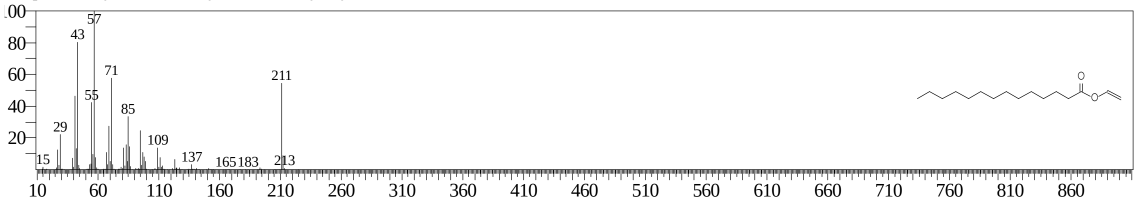

<< Target >>
Line#:25 R.Time:29.075(Scan#:3130) MassPeaks:645
RawMode:Averaged 29.067-29.083(3129-3131) BasePeak:57.05(51263)
BG Mode:Calc. from Peak Group 1 - Event 1 Scan

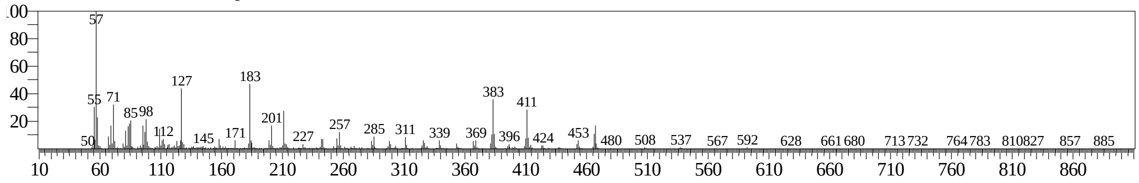

Hit#:1 Entry:109740 Library:NIST20M1.lib
SI:64 Formula:C16H30O2 CAS:5809-91-6 MolWeight:254 RetIndex:1769
CompName:Myristic acid vinyl ester $$ Vinyl myristate #

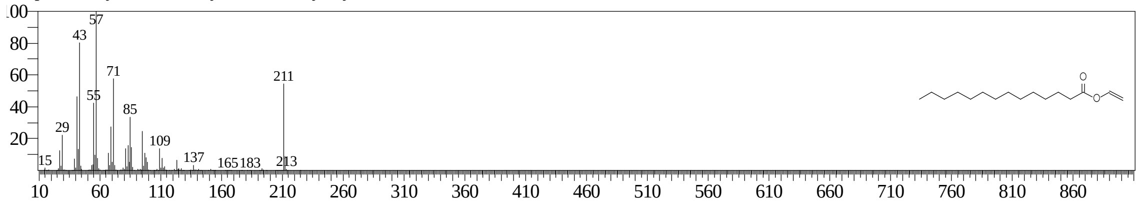

<< Target >>
Line#:26 R.Time:29.417(Scan#:3171) MassPeaks:616
RawMode:Averaged 29.408-29.425(3170-3172) BasePeak:57.05(20666)
BG Mode:Calc. from Peak Group 1 - Event 1 Scan

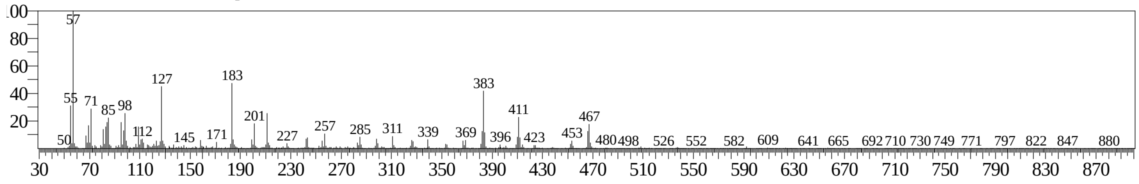

Hit#:1 Entry:247821 Library:NIST20M1.lib
SI:62 Formula:C16H25F7O2 CAS:0-00-0 MolWeight:382 RetIndex:1336
CompName:Dodecanoic acid, 2,2,3,3,4,4,4-heptafluorobutyl ester

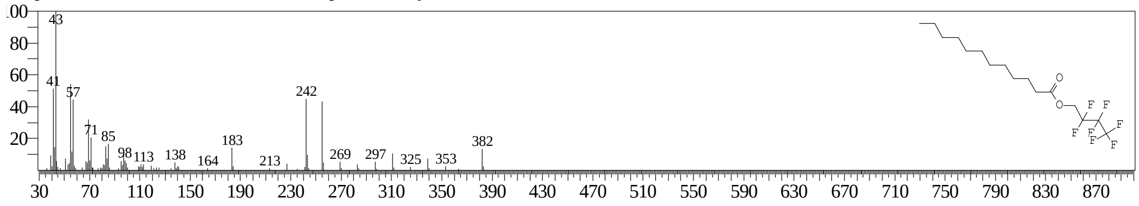

<< Target >>
Line#:27 R.Time:29.975(Scan#:3238) MassPeaks:608
RawMode:Averaged 29.967-29.983(3237-3239) BasePeak:57.05(61958)
BG Mode:Calc. from Peak Group 1 - Event 1 Scan

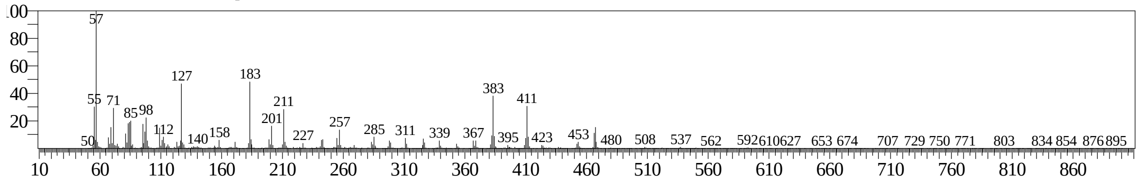

Hit#:1 Entry:109740 Library:NIST20M1.lib
SI:63 Formula:C16H30O2 CAS:5809-91-6 MolWeight:254 RetIndex:1769
CompName:Myristic acid vinyl ester $$ Vinyl myristate #

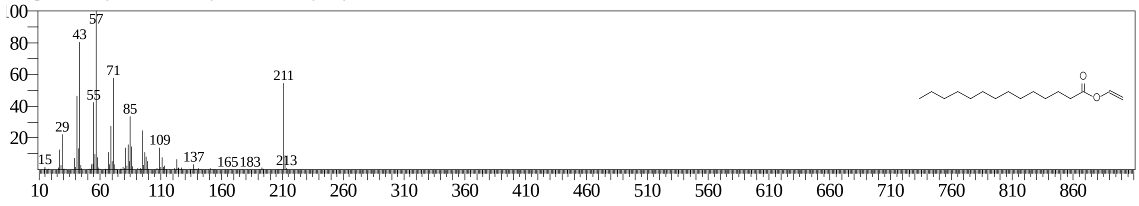

<< Target >>
Line#:28 R.Time:30.767(Scan#:3333) MassPeaks:541
RawMode:Averaged 30.758-30.775(3332-3334) BasePeak:57.05(20303)
BG Mode:Calc. from Peak Group 1 - Event 1 Scan

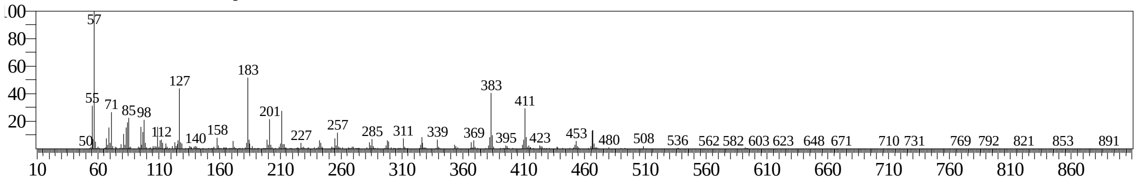

Hit#:1 Entry:79523 Library:NIST20M1.lib
SI:62 Formula:C14H26O2 CAS:2146-71-6 MolWeight:226 RetIndex:1570
CompName:Dodecanoic acid, ethenyl ester $$ Lauric acid, vinyl ester $$ Vinyl laurate $$ Vinyl dodecanoate

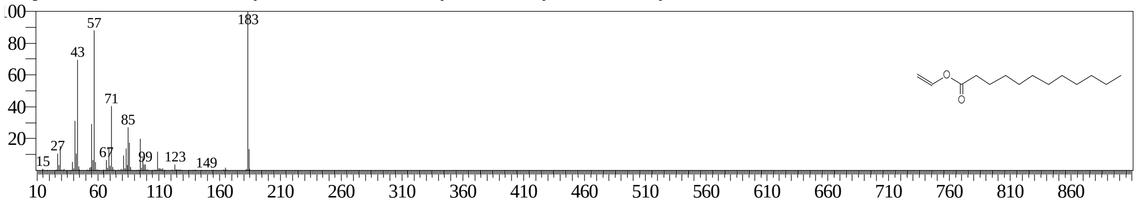

<< Target >>
Line#:29 R.Time:31.275(Scan#:3394) MassPeaks:555
RawMode:Averaged 31.267-31.283(3393-3395) BasePeak:57.05(37542)
BG Mode:Calc. from Peak Group 1 - Event 1 Scan

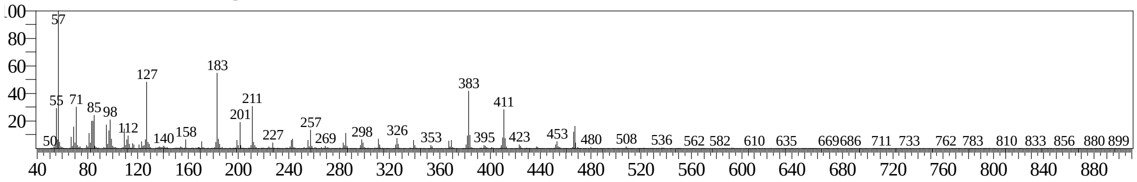

Hit#:1 Entry:94549 Library:NIST20M1.lib
SI:62 Formula:C17H36 CAS:0-00-0 MolWeight:240 RetIndex:1627
CompName:5,5-Diethyltridecane

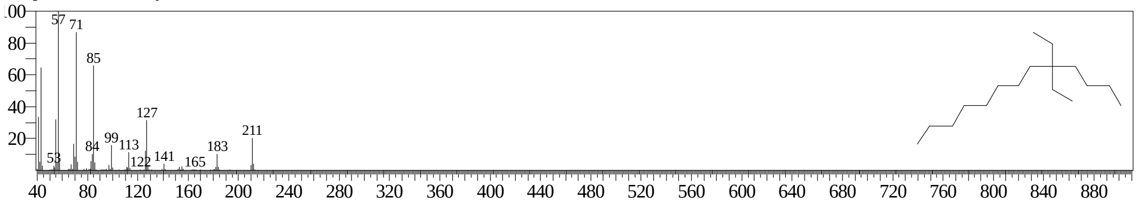

<< Target >>
Line#:30 R.Time:31.883(Scan#:3467) MassPeaks:568
RawMode:Averaged 31.875-31.892(3466-3468) BasePeak:57.05(67246)
BG Mode:Calc. from Peak Group 1 - Event 1 Scan

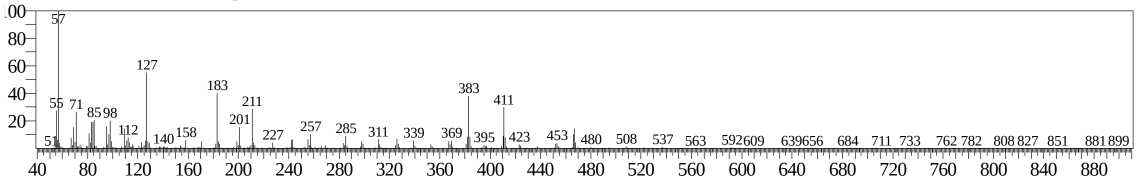

Hit#:1 Entry:94549 Library:NIST20M1.lib
SI:64 Formula:C17H36 CAS:0-00-0 MolWeight:240 RetIndex:1627
CompName:5,5-Diethyltridecane

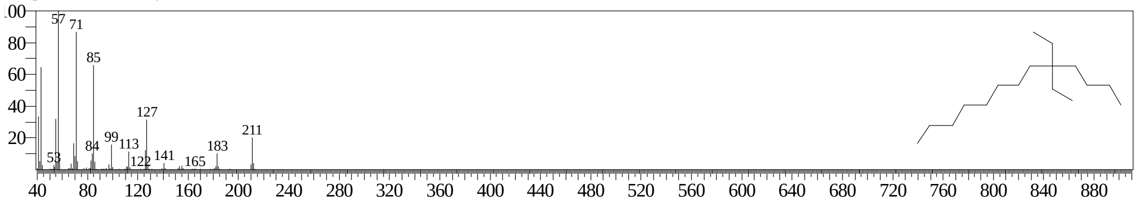

:< Target >>
.ine#:31 R.Time:32.367(Scan#:3525) MassPeaks:439
RawMode:Averaged 32.358-32.375(3524-3526) BasePeak:73.05(960)
3G Mode:Calc. from Peak Group 1 - Event 1 Scan

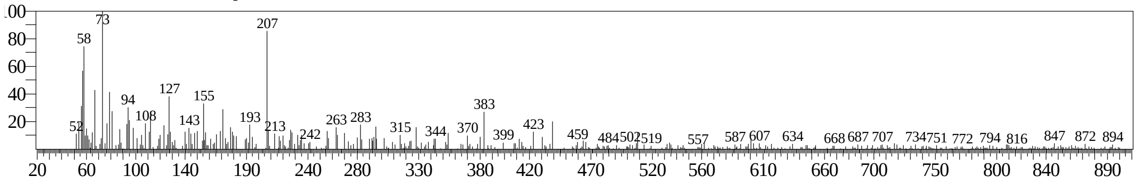

Hit#:1 Entry:102904 Library:NIST20M1.lib
SI:46 Formula:C15H24N2O CAS:3382-84-1 MolWeight:248 RetIndex:2024
CompName:Tetrahydrorhombifoline $$ 1,5-Methano-8H-pyrido[1,2-a][1,5]diazocin-8-one, 3-(3-buten-1-yl)decahydro-, (1S,5R,11aR)- $$ 1,5-Methano-8H-pyri

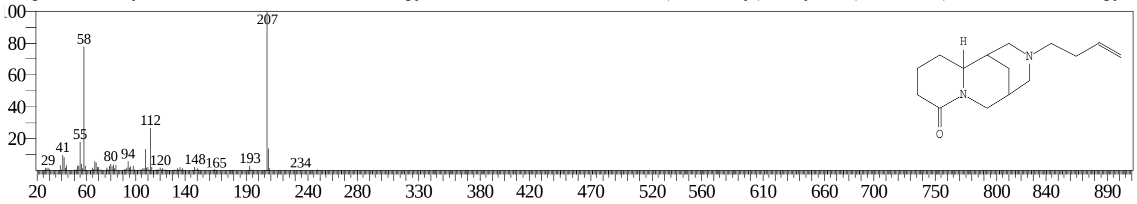

:< Target >>
.ine#:32 R.Time:33.117(Scan#:3615) MassPeaks:499
RawMode:Averaged 33.108-33.125(3614-3616) BasePeak:218.10(6729)
3G Mode:Calc. from Peak Group 1 - Event 1 Scan

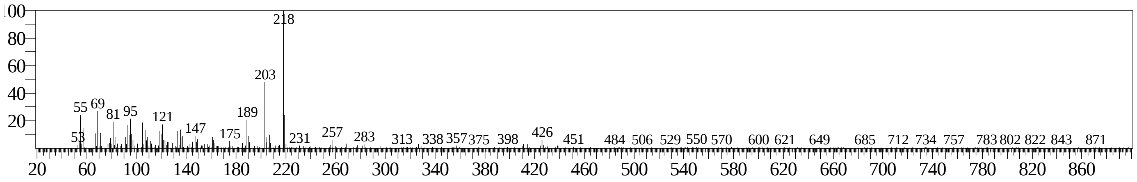

Hit#:1 Entry:257020 Library:NIST20M1.lib
SI:81 Formula:C29H46 CAS:201358-24-9 MolWeight:394 RetIndex:2635
CompName:24-Noroleana-3,12-diene $$ (4aR,6aS,6bR,12aS,12bR,14bS)-2,2,4a,6a,6b,9,12a-Heptamethyl-1,2,3,4,4a,5,6,6a,6b,7,8,8a,11,12,12a,12b,13,14b-oc

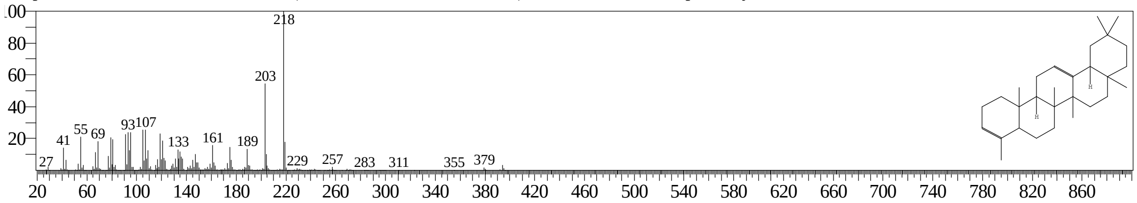

:< Target >>
.ine#:33 R.Time:33.667(Scan#:3681) MassPeaks:480
RawMode:Averaged 33.658-33.675(3680-3682) BasePeak:176.05(24714)
3G Mode:Calc. from Peak Group 1 - Event 1 Scan

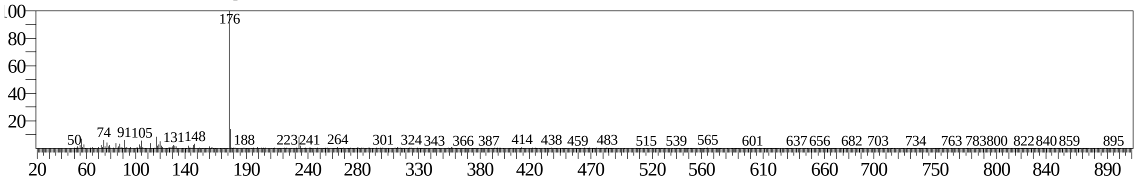

Hit#:1 Entry:179051 Library:NIST20M1.lib
SI:77 Formula:C19H28BNO2 CAS:0-00-0 MolWeight:313 RetIndex:0
CompName:Benzoic acid, p-(diethylamino)-, 9-borabicyclo[3.3.1]non-9-yl ester $$ 4-[(9-Borabicyclo[3.3.1]non-9-yloxy)carbonyl]-N,N-diethylaniline #

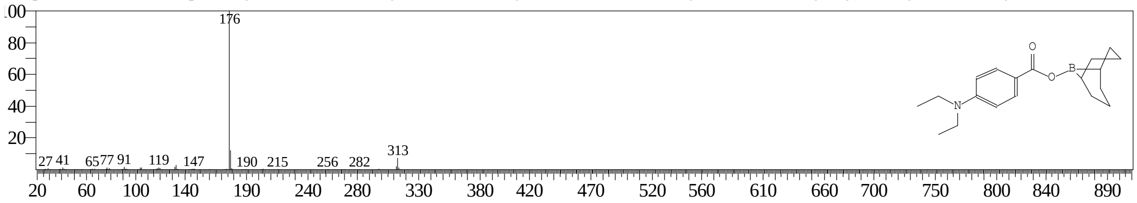

<< Target >>
Line#:34 R.Time:34.283(Scan#:3755) MassPeaks:521
RawMode:Averaged 34.275-34.292(3754-3756) BasePeak:81.05(5387)
BG Mode:Calc. from Peak Group 1 - Event 1 Scan

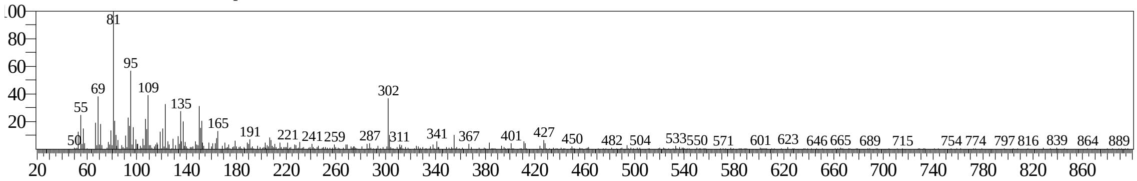

Hit#:1 Entry:231830 Library:NIST20M1.lib
SI:82 Formula:C22H34O4 CAS:50656-65-0 MolWeight:362 RetIndex:2531
CompName:Rotundifuran $$ 1,4-Naphthalenediol, 1-[2-(3-furanyl)ethyl]decahydro-2,5,5,8a-tetramethyl-, 4-acetate, (1R,2R,4R,4aS,8aS)- $$ 1,4-Naphthalenedi

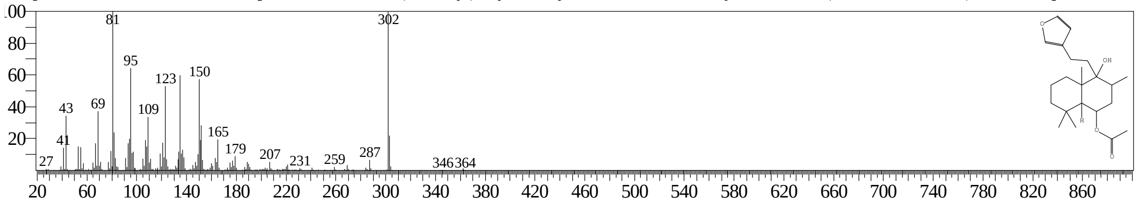

<< Target >>
Line#:35 R.Time:35.258(Scan#:3872) MassPeaks:526
RawMode:Averaged 35.250-35.267(3871-3873) BasePeak:99.10(9708)
BG Mode:Calc. from Peak Group 1 - Event 1 Scan

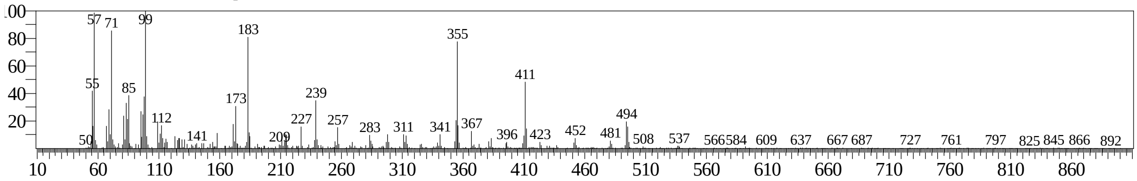

Hit#:1 Entry:209815 Library:NIST20M1.lib
SI:60 Formula:C20H36O4 CAS:0-00-0 MolWeight:340 RetIndex:2224
CompName:Fumaric acid, 2-methylpentyl dec-2-yl ester

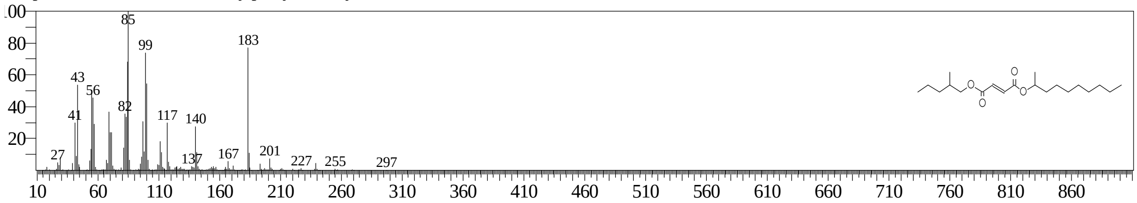

<< Target >>
Line#:36 R.Time:35.600(Scan#:3913) MassPeaks:445
RawMode:Averaged 35.592-35.608(3912-3914) BasePeak:57.05(1261)
BG Mode:Calc. from Peak Group 1 - Event 1 Scan

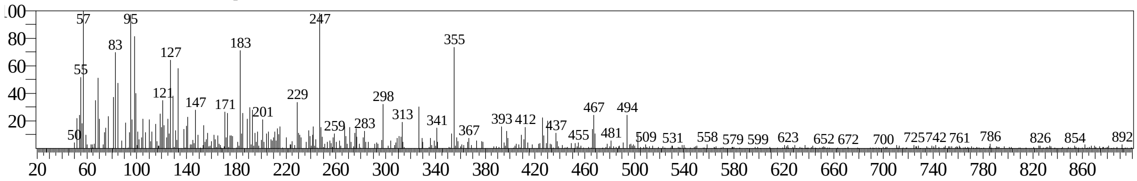

Hit#:1 Entry:139655 Library:NIST20M1.lib
SI:48 Formula:C18H32O2 CAS:0-00-0 MolWeight:280 RetIndex:1976
CompName:Undec-10-ynoic acid, heptyl ester

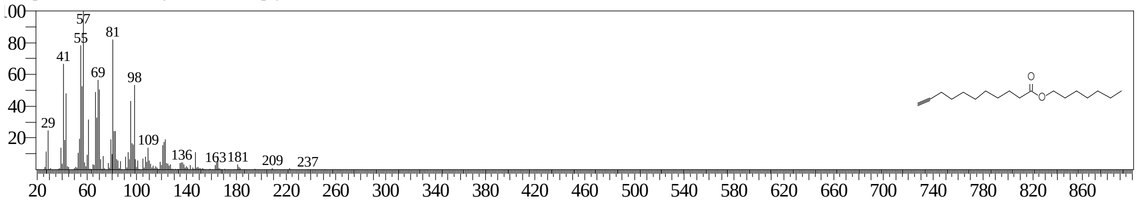

<< Target >>
.ine#:37 R.Time:36.433(Scan#:4013) MassPeaks:464
RawMode:Averaged 36.425-36.442(4012-4014) BasePeak:218.05(1212)
BG Mode:Calc. from Peak Group 1 - Event 1 Scan

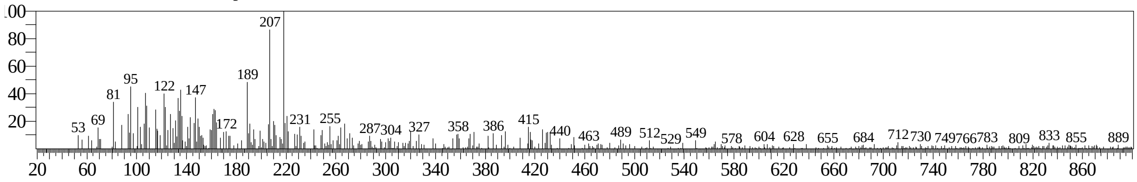

Hit#:1 Entry:137347 Library:NIST20M1.lib
SI:55 Formula:C17H26O3 CAS:1005273-98-2 MolWeight:278 RetIndex:2018
CompName:Acetic acid, 3-hydroxy-7-isopropenyl-1,4a-dimethyl-2,3,4,4a,5,6,7,8-octahydronaphthalen-2-yl ester $$ 3-Hydroxy-7-isopropenyl-1,4a-dimethyl-2,3

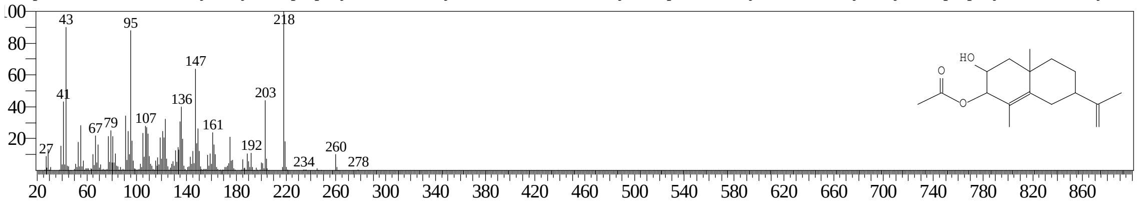

<< Target >>
.ine#:38 R.Time:37.033(Scan#:4085) MassPeaks:380
RawMode:Averaged 37.025-37.042(4084-4086) BasePeak:74.05(2693)
BG Mode:Calc. from Peak Group 1 - Event 1 Scan

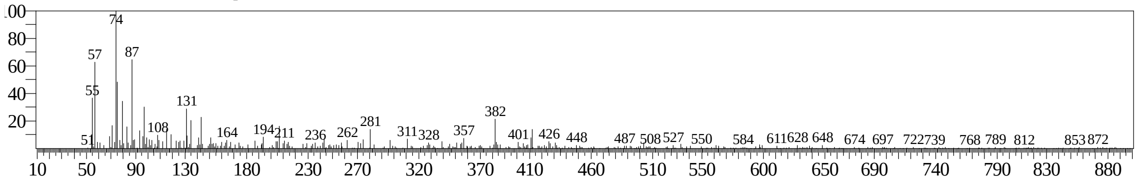

Hit#:1 Entry:248690 Library:NIST20M1.lib
SI:63 Formula:C25H50O2 CAS:2442-49-1 MolWeight:382 RetIndex:2674
CompName:Tetracosanoic acid, methyl ester $$ Methyl lignocerate $$ Methyl tetracosanoate $$ Lignoceric acid methyl ester

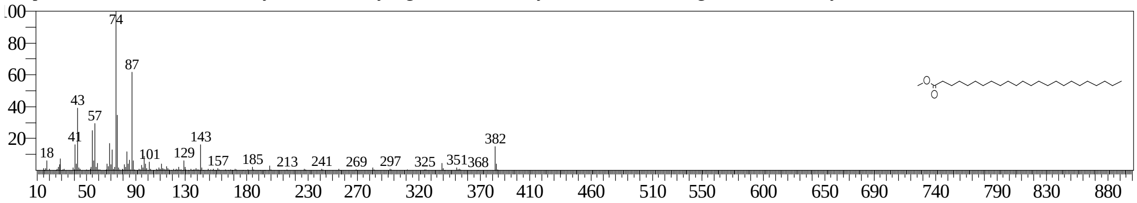

<< Target >>
.ine#:39 R.Time:37.267(Scan#:4113) MassPeaks:440
RawMode:Averaged 37.258-37.275(4112-4114) BasePeak:280.90(726)
BG Mode:Calc. from Peak Group 1 - Event 1 Scan

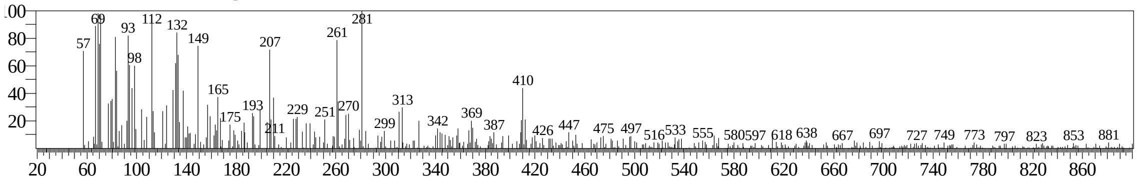

Hit#:1 Entry:254486 Library:NIST20M1.lib
SI:49 Formula:C24H38O4 CAS:6422-86-2 MolWeight:390 RetIndex:2704
CompName:1,4-Benzenedicarboxylic acid, bis(2-ethylhexyl) ester $$ Terephthalic acid, bis(2-ethylhexyl)ester $$ Bis(2-ethylhexyl) terephthalate $$ Kodaflex dc

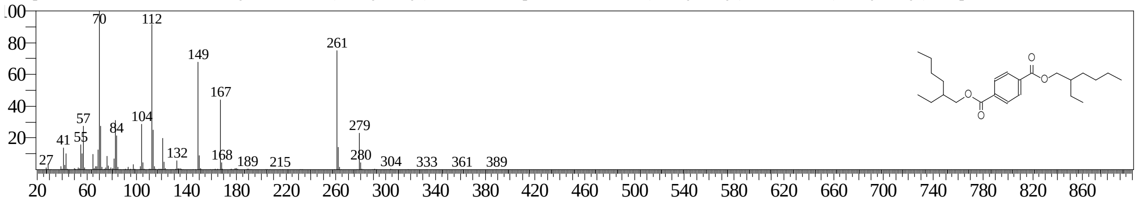

<< Target >>
Line#:40 R.Time:38.233(Scan#:4229) MassPeaks:482
RawMode:Averaged 38.225-38.242(4228-4230) BasePeak:69.10(74887)
BG Mode:Calc. from Peak Group 1 - Event 1 Scan

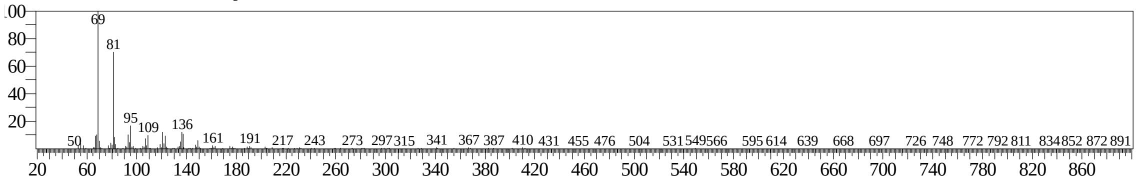

Hit#:1 Entry:212569 Library:NIST20M1.lib
SI:90 Formula:C25H42 CAS:75581-03-2 MolWeight:342 RetIndex:2432
CompName:2,6,10,14,18-Pentamethyl-2,6,10,14,18-eicosapentaene $$ (6E,10E,14E,18E)-2,6,10,14,18-Pentamethyl-2,6,10,14,18-icosapentaene #

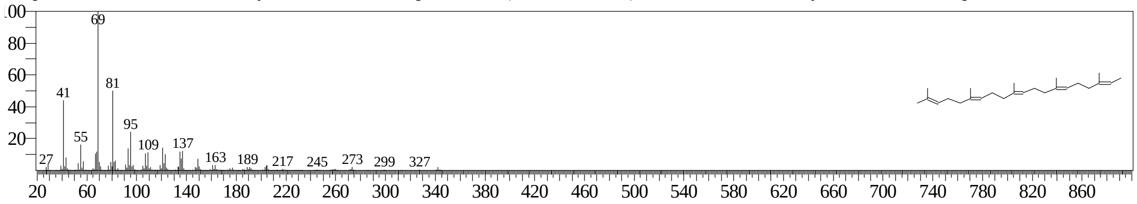

<< Target >>
Line#:41 R.Time:39.042(Scan#:4326) MassPeaks:565
RawMode:Averaged 39.033-39.050(4325-4327) BasePeak:124.10(3116)
BG Mode:Calc. from Peak Group 1 - Event 1 Scan

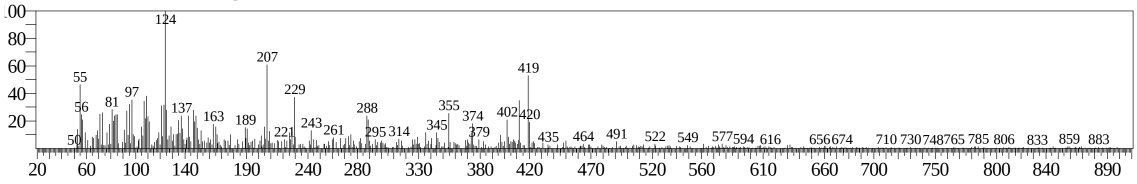

Hit#:1 Entry:89914 Library:NIST20M1.lib
SI:61 Formula:C15H24O2 CAS:61050-91-7 MolWeight:236 RetIndex:1626
CompName:Spiro[4.5]decan-7-one, 1,8-dimethyl-8,9-epoxy-4-isopropyl-

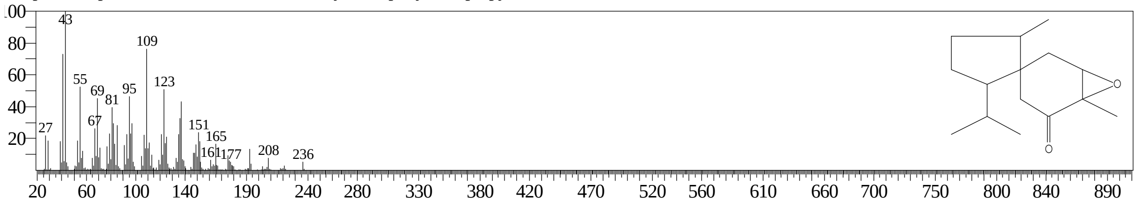

<< Target >>
Line#:42 R.Time:40.100(Scan#:4453) MassPeaks:493
RawMode:Averaged 40.092-40.108(4452-4454) BasePeak:81.05(1601)
BG Mode:Calc. from Peak Group 1 - Event 1 Scan

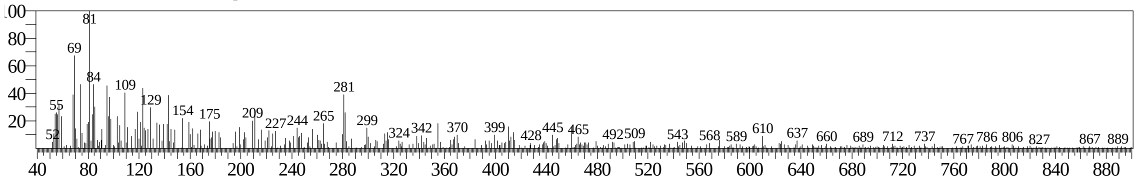

Hit#:1 Entry:193693 Library:NIST20M1.lib
SI:60 Formula:C18H30O5 CAS:108533-24-0 MolWeight:326 RetIndex:2021
CompName:2-Butyloxycarbonyloxy-1,1,10-trimethyl-6,9-epidioxydecalin

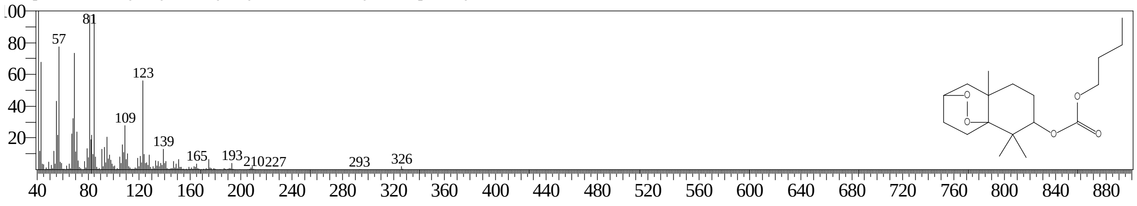

<< Target >>
Line#:43 R.Time:40.683(Scan#:4523) MassPeaks:496
RawMode:Averaged 40.675-40.692(4522-4524) BasePeak:416.10(35391)
BG Mode:Calc. from Peak Group 1 - Event 1 Scan

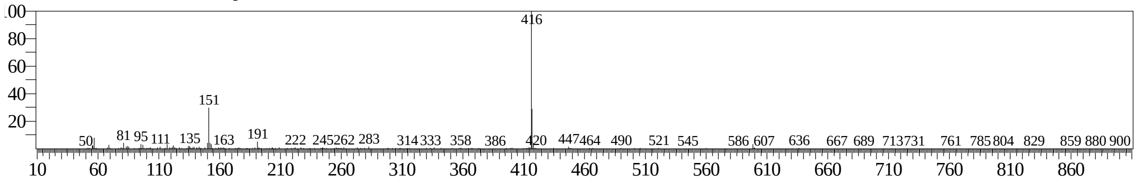

Hit#:1 Entry:107122 Library:NIST20M1.lib
SI:44 Formula:C14H24N2O2 CAS:0-00-0 MolWeight:252 RetIndex:2043
CompName:1-(Cyclopropylcarbonyl)-3-piperidinamine, N-trimethylacetyl-

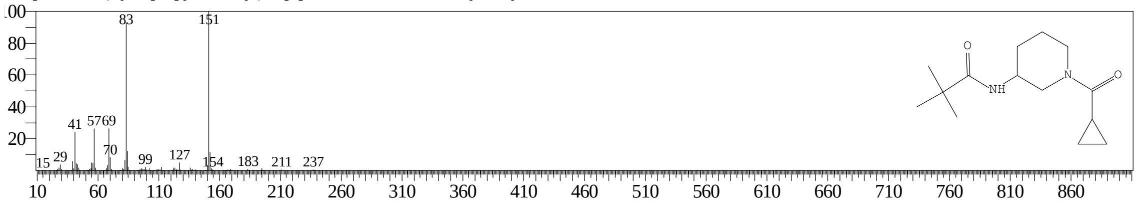

<< Target >>
Line#:44 R.Time:41.767(Scan#:4653) MassPeaks:484
RawMode:Averaged 41.758-41.775(4652-4654) BasePeak:295.00(2254)
BG Mode:Calc. from Peak Group 1 - Event 1 Scan

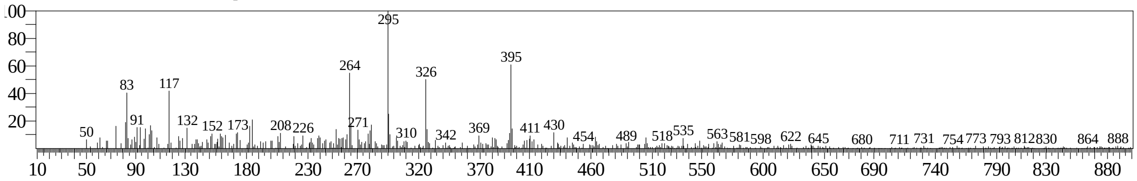

Hit#:1 Entry:193373 Library:NIST20M1.lib
SI:40 Formula:C17H14N2O3S CAS:20863-09-6 MolWeight:326 RetIndex:3080
CompName:N-(2-Hydroxyethyl)-4-methylthio-1,2-carbazoledicarboximide $$ 2-(2-Hydroxyethyl)-5-(methylsulfanyl)pyrrolo[3,4-a]carbazole-1,3(2H,10H)-dione

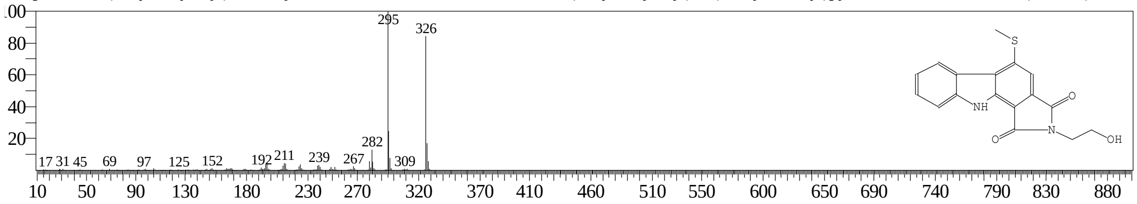

<< Target >>
Line#:45 R.Time:42.367(Scan#:4725) MassPeaks:404
RawMode:Averaged 42.358-42.375(4724-4726) BasePeak:151.05(4399)
BG Mode:Calc. from Peak Group 1 - Event 1 Scan

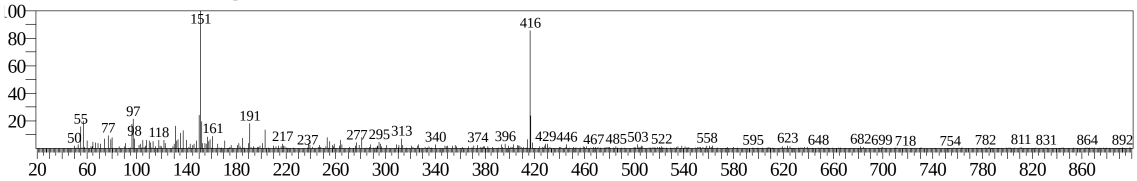

Hit#:1 Entry:168652 Library:NIST20M1.lib
SI:50 Formula:C20H32O2 CAS:0-00-0 MolWeight:304 RetIndex:2267
CompName:4-Ethylbenzoic acid, undecyl ester $$ Undecyl 4-ethylbenzoate #

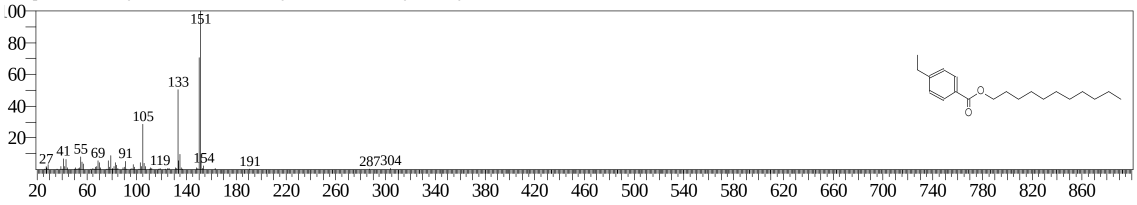

<< Target >>
Line#:46 R.Time:42.800(Scan#:4777) MassPeaks:490
RawMode:Averaged 42.792-42.808(4776-4778) BasePeak:81.05(2098)
BG Mode:Calc. from Peak Group 1 - Event 1 Scan

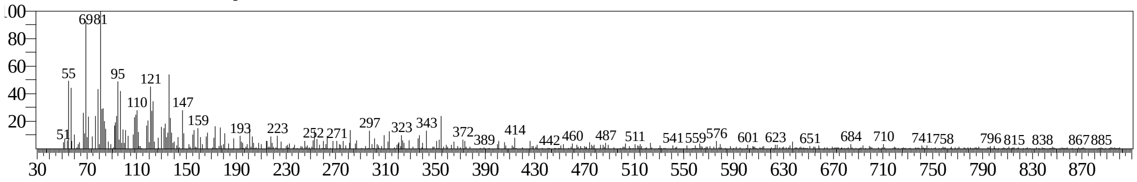

Hit#:1 Entry:75401 Library:NIST20M1.lib
SI:70 Formula:C15H26O CAS:35727-45-8 MolWeight:222 RetIndex:1555
CompName:Cyclohexanol, 3-ethenyl-3-methyl-2-(1-methylethenyl)-6-(1-methylethyl)-, [1R-(1.alpha.,2.alpha.,3.beta.,6.alpha.)]- $$ Shyobunol $$ 1.beta.-Isopro

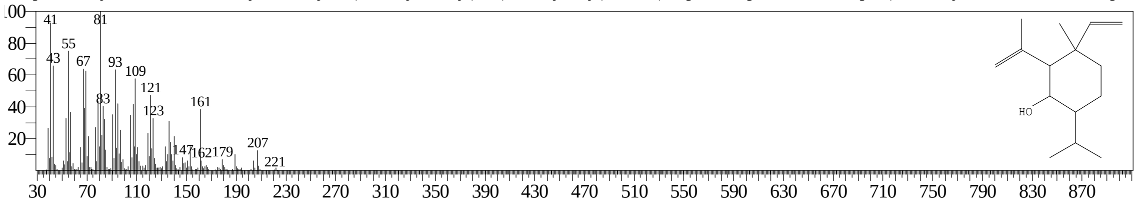

<< Target >>
Line#:47 R.Time:43.100(Scan#:4813) MassPeaks:441
RawMode:Averaged 43.092-43.108(4812-4814) BasePeak:79.05(966)
BG Mode:Calc. from Peak Group 1 - Event 1 Scan

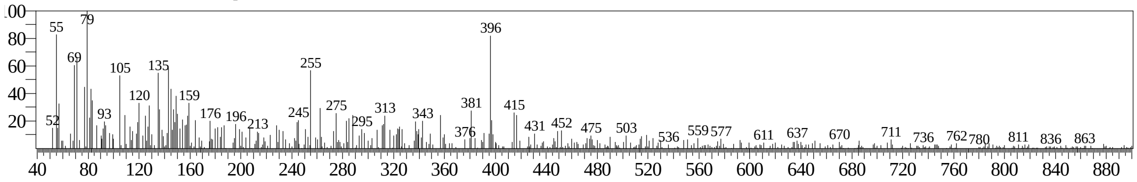

Hit#:1 Entry:258406 Library:NIST20M1.lib
SI:56 Formula:C29H48 CAS:4970-37-0 MolWeight:396 RetIndex:2525
CompName:Stigmasta-3,5-diene $$ (8S,9S,10R,13R,14S,17R)-17-((2R)-5-Ethyl-6-methylheptan-2-yl)-10,13-dimethyl-2,7,8,9,10,11,12,13,14,15,16,17-dodecal

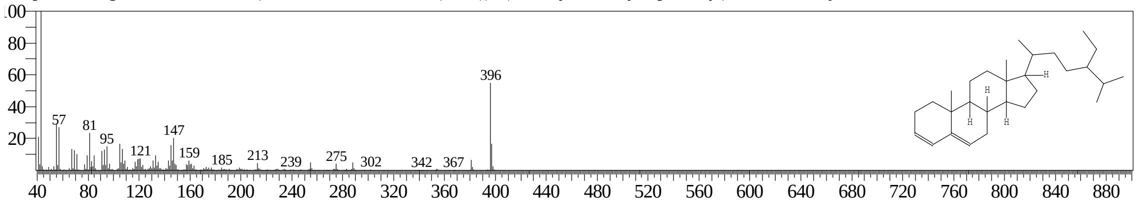

<< Target >>
Line#:48 R.Time:43.967(Scan#:4917) MassPeaks:499
RawMode:Averaged 43.958-43.975(4916-4918) BasePeak:430.10(30032)
BG Mode:Calc. from Peak Group 1 - Event 1 Scan

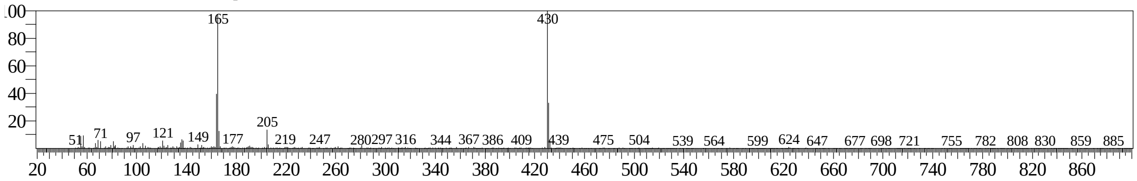

Hit#:1 Entry:253147 Library:NIST20M1.lib
SI:59 Formula:C26H44O2 CAS:0-00-0 MolWeight:388 RetIndex:2863
CompName:Benzoic acid, 4-propyl-, hexadecyl ester

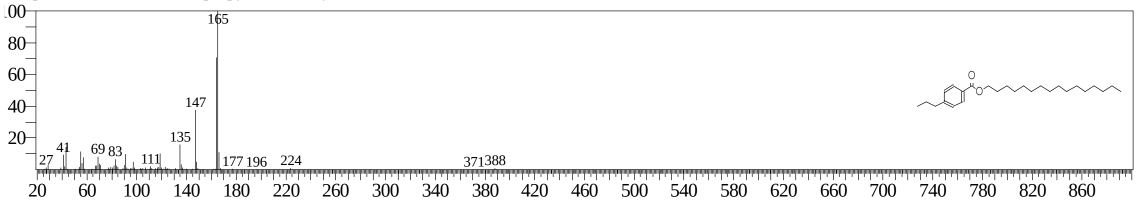

<< Target >>
Line#:49 R.Time:44.458(Scan#:4976) MassPeaks:515
RawMode:Averaged 44.450-44.467(4975-4977) BasePeak:69.10(2032)
BG Mode:Calc. from Peak Group 1 - Event 1 Scan

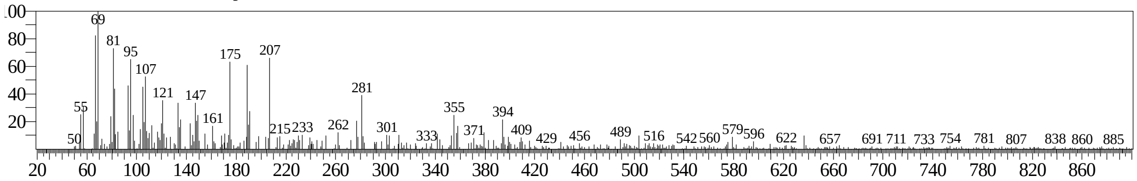

Hit#:1 Entry:57219 Library:NIST20M1.lib
SI:65 Formula:C15H24 CAS:0-00-0 MolWeight:204 RetIndex:1475
CompName:Cycloheptane, 4-methylene-1-methyl-2-(2-methyl-1-propen-1-yl)-1-vinyl- $$ 1-Methyl-4-methylene-2-(2-methyl-1-propenyl)-1-vinylcycloheptane #

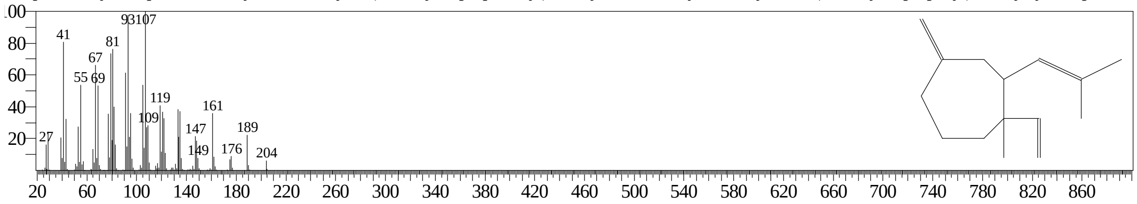

<< Target >>
Line#:50 R.Time:45.133(Scan#:5057) MassPeaks:477
RawMode:Averaged 45.125-45.142(5056-5058) BasePeak:69.05(1009)
BG Mode:Calc. from Peak Group 1 - Event 1 Scan

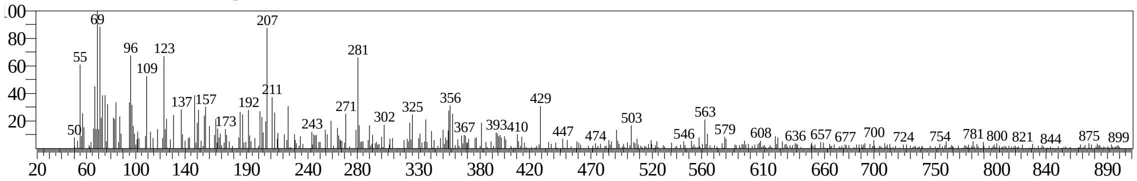

Hit#:1 Entry:175540 Library:NIST20M1.lib
SI:48 Formula:C20H38O2 CAS:0-00-0 MolWeight:310 RetIndex:2257
CompName:8,15-Labdanediol $$ (2R)-1-(5-Hydroxy-3-methylpentyl)-2,5,5,8a-tetramethyldecahydronaphthalen-2-ol

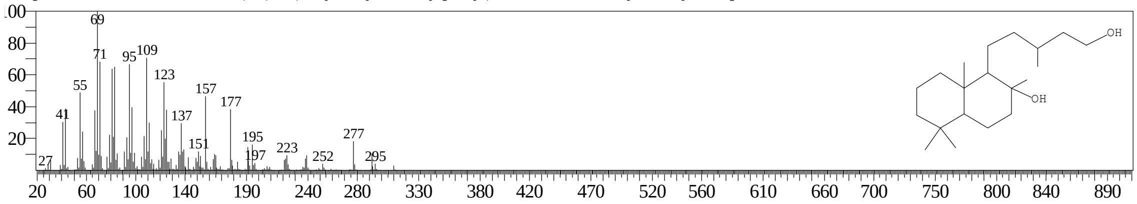

<< Target >>
Line#:51 R.Time:46.167(Scan#:5181) MassPeaks:441
RawMode:Averaged 46.158-46.175(5180-5182) BasePeak:79.05(753)
BG Mode:Calc. from Peak Group 1 - Event 1 Scan

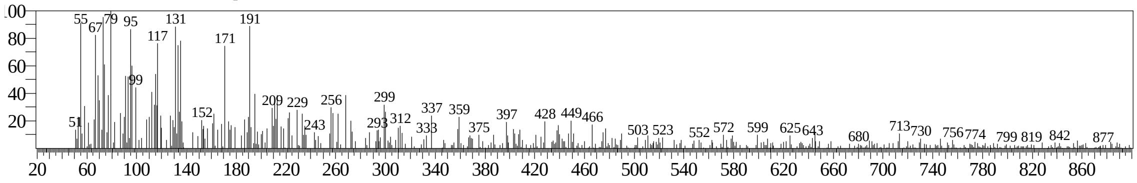

Hit#:1 Entry:242516 Library:NIST20M1.lib
SI:46 Formula:C23H38O2Si CAS:0-00-0 MolWeight:374 RetIndex:2425
CompName:Eicosapentaenoic Acid, TMS derivative $$ cis-5,8,11,14,17-Eicosapentaenoic acid, trimethylsilyl ester

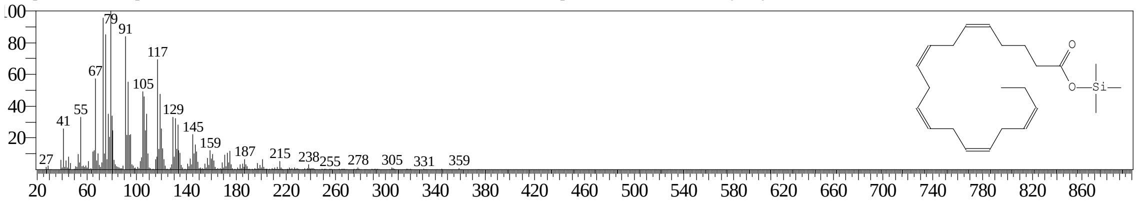

<< Target >>
ine#:52 R.Time:46.900(Scan#:5269) MassPeaks:441
RawMode:Averaged 46.892-46.908(5268-5270) BasePeak:57.05(2318)
G Mode:Calc. from Peak Group 1 - Event 1 Scan

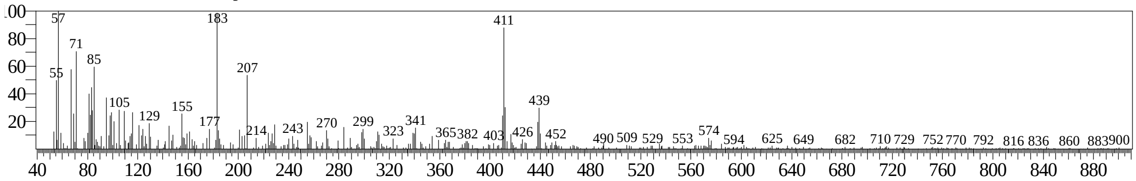

it#:1 Entry:81727 Library:NIST20M1.lib
I:55 Formula:C14H28O2 CAS:108682-08-2 MolWeight:228 RetIndex:1826
ompName:Cyclododecanol, 1-(methoxymethyl)- $$ 1-(Methoxymethyl)cyclododecanol #

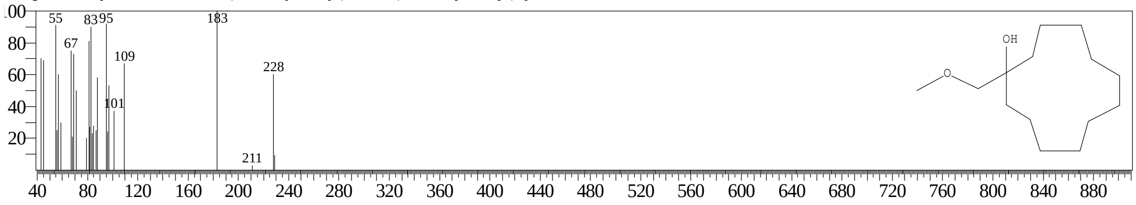

<< Target >>
ine#:53 R.Time:47.167(Scan#:5301) MassPeaks:465
RawMode:Averaged 47.158-47.175(5300-5302) BasePeak:83.05(2786)
G Mode:Calc. from Peak Group 1 - Event 1 Scan

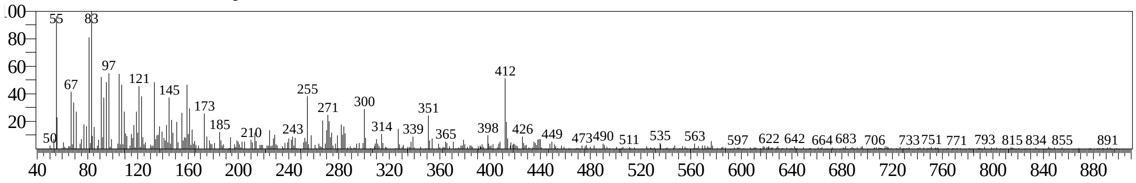

it#:1 Entry:226618 Library:NIST20M1.lib
I:72 Formula:C25H40O CAS:57597-10-1 MolWeight:356 RetIndex:2470
ompName:26,27-Dinorcholesta-5,22-dien-3-ol, (3.beta.,22E)-

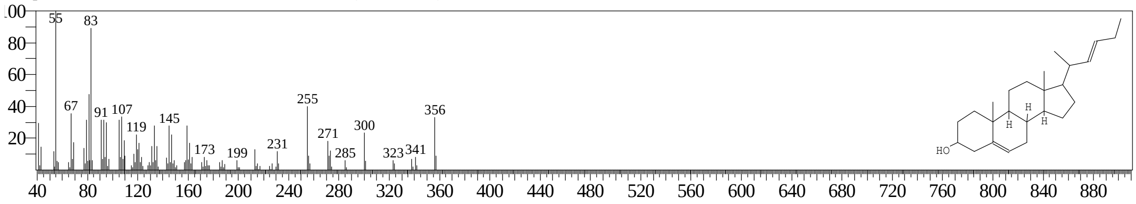

<< Target >>
ine#:54 R.Time:47.775(Scan#:5374) MassPeaks:463
RawMode:Averaged 47.767-47.783(5373-5375) BasePeak:57.05(2689)
G Mode:Calc. from Peak Group 1 - Event 1 Scan

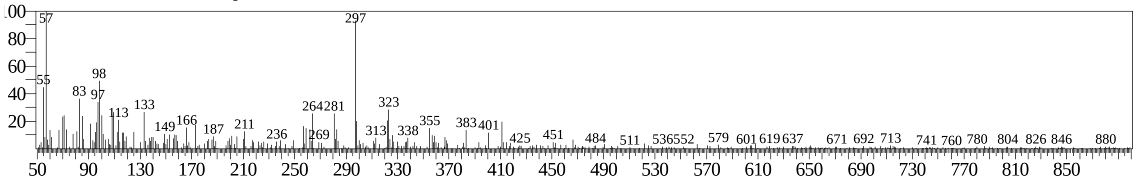

it#:1 Entry:178128 Library:NIST20M1.lib
I:57 Formula:C20H40O2 CAS:0-00-0 MolWeight:312 RetIndex:2101
ompName:14-Methyl-hexadecane-1,2-diol, isopropylidene

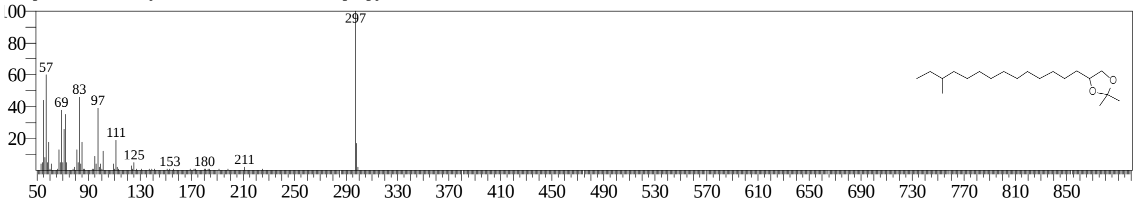

Supplement: Supplementary file 2 — Supplementary Material 2 [file 13002_2026_896_MOESM2_ESM.pdf]
